# From Squid to Mammals with the HH Model through the *Na_v_* Channels’ Half-Activation-Voltage Parameter

**DOI:** 10.1371/journal.pone.0143570

**Published:** 2015-12-02

**Authors:** Nedialko I. Krouchev, Frank Rattay, Mohamad Sawan, Alain Vinet

**Affiliations:** 1 Polystim Neurotechnologies, Ecole Polytechnique, Montreal (Quebec), Canada; 2 Institute for Analysis and Scientific Computing, University of Technology, Vienna, Austria; 3 Institut de Genie Biomedical, Universite de Montreal, Montreal (Quebec), Canada; Georgia State University, UNITED STATES

## Abstract

**The model family** analyzed in this work stems from the classical Hodgkin-Huxley model (HHM). for a single-compartment (space-clamp) and continuous variation of the voltage-gated sodium channels (*Na*_*v*_) half-activation-voltage parameter Δ*V*_1/2_, which controls the window of sodium-influx currents. Unlike the baseline HHM, its parametric extension exhibits a richer multitude of dynamic regimes, such as multiple fixed points (FP’s), bi- and multi-stability (coexistence of FP’s and/or periodic orbits). Such diversity correlates with a number of functional properties of excitable neural tissue, such as the capacity or not to evoke an action potential (AP) from the resting state, by applying a minimal *absolute rheobase* current amplitude. **The utility** of the HHM rooted in the giant squid for the descriptions of the mammalian nervous system is of topical interest. We conclude that the model’s fundamental principles are still valid (up to using appropriate parameter values) for warmer-blooded species, without a pressing need for a substantial revision of the mathematical formulation. We demonstrate clearly that the continuous variation of the Δ*V*_1/2_ parameter comes close to being equivalent with recent HHM ‘optimizations’. **The neural dynamics** phenomena described here are nontrivial. The model family analyzed in this work contains the classical HHM as a special case. The validity and applicability of the HHM to mammalian neurons can be achieved by picking the appropriate Δ*V*_1/2_ parameter in a significantly broad range of values. For such large variations, in contrast to the classical HHM, the *h* and *n* gates’ dynamics may be *uncoupled* - i.e. the *n* gates may no longer be considered in mere linear correspondence to the *h* gates. Δ*V*_1/2_ **variation** leads to a multitude of dynamic regimes—e.g. models with either 1 fixed point (FP) or with 3 FP’s. These may also coexist with stable and/or unstable periodic orbits. Hence, depending on the initial conditions, the system may behave as either purely excitable or as an oscillator. Δ*V*_1/2_ variation leads to significant changes in the metabolic efficiency of an action potential (AP). Lower Δ*V*_1/2_ values yield a larger range of AP response frequencies, and hence provide for more flexible neural coding. Such lower values also contribute to faster AP conduction velocities along neural fibers of otherwise comparable-diameter. The 3 FP case brings about an *absolute* rheobase current. In comparison in the classical HHM the rheobase current is only relative - i.e. excitability is lost after a *finite* amount of elapsed stimulation time. Lower Δ*V*_1/2_ values translate in lower threshold currents from the resting state.

## Introduction

The Hodgkin-Huxley (HH) model recently turned 60 [1, 2]. It has been the only widely used prototype for *microscopic* single-compartment models [3]. Other popular models (e.g. the leaky integrate’n fire) do not contend for either detailed and type-specific descriptions of single neurons, or even less of their parts.

More recently, related to the detailed microscopic reconstructions, multi-compartmental neuronal models are built of electrically coupled HH-type compartments. The model has eloquently proven its *quantitative* capacity to convey an explanatory power to relatively simple neuron models, which account for more and more experimental findings [3]. Yet it has also been criticized, re-parameterized and subjected to multiple attempts to find it incomplete or inefficient [4, 5]. Importantly, the HH model (Table 1 introduces all the commonly used abbreviations) simplicity was blamed, while critics sometimes omitted essential properties themselves [6]. The model’s very applicability to mammalian neurons—with respect to metabolic requirements and flexibility of encoding, has been closely reexamined [2, 7, 8].

**Table 1:** Commonly used abbreviations.

Symbol	Description
0D	zero-dimensional, i.e. single-compartment or space clamp models; whose spatial extents are confined to a point
1D	cable-like, multi-compartment spatial structure; homo-morphic to line
2D etc.	two- or more dimensional, refers to the number of states that describe the excitable system’s dynamics
AP	Action potential
B.D.	bifurcation diagram
BVDP	the Bonhoeffer-Van der Pol oscillator-dynamics model; also known as the Fitzhugh-Nagumo model
ES	Electrical stimulation
FP	Fixed point of system dynamics → vanishing derivative(s)
HH or HHM	Hodgkin and Huxley’s [model of excitable membranes]
ML or MLM	Morris and Lecar’s [model of the barnacle giant muscle fiber]
ODE	Ordinary Differential equation; see also PDE
PDE	Differential equation involving partial derivatives; see also ODE
PO	Periodic orbit (or limit cycle)—Closed (starting and ending at the same point in phase space) dynamic trajectory The period of the PO may be finite or →∞. In the latter case it may be a hetero- or homo-clinic (starting and ending at either two distinct FP’s, or the same single *half-stable* FP, respectively)
PTC	phase transition curve
RGC	retinal ganglion cells
RHS	right-hand side
SD	*strength-duration* [curve]
S.T.	such that
W.R.T.	with respect to

An unifying parametric framework is proposed in this work to systematically examine the impact of *Na*_*v*_ subtypes’ distributions on neuronal dynamics, and thence on excitability and refractoriness.

We systematically explore the influence of parameter variation on the fundamental biophysical properties of voltage-gated sodium (*Na*_*v*_) channels, within an HH-type modeling framework. The *Na*_*v*_ channels produce large and fast membrane currents essential in the generation and propagation of action potentials in excitable tissues. They contain a transmembrane alpha subunit which forms the channel pore. The latter subunit’s genetic expression proved sufficient for the expression of whole functional channels of a given subtype [9]. Hence *Na* ^+^ channel nomenclature follows closely that of their alpha subunit (in this work we use the *Na*_*v*1.*X*_ notation). With the help of gene engineering and selective expression of specific *Na*_*v*1.*X*_ channels, significant experimental evidence has been accumulated on their (in)activation and localization properties [10–12].

Relatively little is known about the ways in which the different *Na* ^+^ channel subtypes are distributed and expressed toward functional axons in either a developmental or mature stage [13, 14]. An exceptional wealth of evidence comes from epilepsy research [15–20], where *Na* ^+^ channel mutations have been associated with either gain-of-function or loss-of-function effects [19]—i.e. increased or decreased neuronal excitability in either excitatory or inhibitory populations (e.g. GABA interneurons or Purkinje cells). The *Na*_*v*1.1_ subtype, which is hypothesized to undergo such mutations, is also involved in an important developmental aspect. Namely, it gradually replaces the *low threshold* *Na*_*v*1.3_ subtype—which is only expressed during early development or excitable-tissue injury [21]. Interestingly, both the *Na*_*v*1.1_ and *Na*_*v*1.3_ subtypes are encoded on chromosome 2*q*24. It may also be tempting to speculate that the easily excitable *Na*_*v*1.3_ subtype is desirable during large neural network connectivity formation, but would lead to dynamic stability issues in an adult highly active and interconnected brain.

It is known that action-potentials (AP) are primarily initiated close to the *axon hillock* [22]. The cerebral cortex is densely packed with an estimated 50,000 neurons/*mm* ^3^ and at least 100 times as many neural processes [23]. A recent study [24] used HH models of two cortex-specific *Na*_*v*1.2_ and *Na*_*v*1.6_ subtypes. *Na*_*v*1.2_ sustained AP propagation, while *Na*_*v*1.6_ activation at lower membrane voltage (*V*) values contributed to AP initiation. The same *Na*_*v*_ channel subtypes were then used in a multi-compartmental HH model of a neuron with a single dendrite, soma and axon morphology along a straight axis [25], which robustly interpreted and predicted the clinical effects of intra-cortical micro-stimulation (ICMS), following up on previous work on the subject [26]. It was found that AP initiation in the *axon initial segment* (AIS) was more likely and required lower stimulation current. Moreover, this was attributed to the higher density of the *Na*_*v*1.6_ subtype.

Benefitting from a detailed exploration of the literature on experimentally observed *Na*_*v*_ channel properties in the central nervous system (CNS), we address the *parameter* *V*_1/2_ controlling the membrane voltage *V* at which the *Na* ^+^ conductance attains its half-maximal value. This parameter has a direct impact on a number of fundamental properties. Some of these are straightforward to demonstrate from first biophysical principles. Others required subsequent bifurcation and phase-plane analysis, which are only enabled by models with just a few parameters. Nonetheless, this reductionist approach provides useful generalizations about key aspects under investigation—such as the HH model limits of metabolic efficiency or encoding. We do not claim that the model presented here is a definitive one, or that the different *Na*_*v*_ subtypes are *only* due to simple shifts in *V*_1/2_. Nature has access to many complex molecular structures and there may be restrictions we are unaware of. Hence, overly simplistic models may fail to explain some observations. The proper model type depends on its goals and phenomenological scale [3]. Nonetheless, simple models can generate useful predictions and are easy to incorporate as building blocks in novel more elaborate paradigms. Thus, we use our parametric framework—within a physiological *V*_1/2_ range—to address metabolic energy savings and other desirable properties such as fast AP transmission or large frequency-encoding capacity. We provide a comprehensive excitability-dynamics description of the *Na*_*v*_ subtypes similar to the ones expressed in the mammalian brain (*Na*_*v*1.*X*_ with *X* = 1, 2, or 6). Such analysis over a range of continuous *V*_1/2_ variations seems in order, especially given that the *V*_1/2_ values reported by different authors vary significantly [2, 10–12, 15, 17–21, 27–29]. assuming that one (and only) given *Na*_*v*1.*X*_ subtype is expressed in the host cell, on which whole-cell voltage clamping techniques are applied.

Very recent experimental and computational work on the molecular and functional effects of brain trauma points in quite a similar direction. Namely that *Na*_*v*_ channels undergo irreversible hyperpolarizing *V*_1/2_ shifts [30, 31]. This may for example lead to ectopic excitability and propagation in damaged axons [32].

The presentation is laid out as follows:

The next section (Methods) presents the model details and key assumptions.

In a structured form the Results section defines a fundamental ensemble of related key biophysical properties of the parameterized single-compartment HH model, such as metabolic efficiency and excitability. The theoretical analysis presents the bifurcation structure as a function of the Δ*V*_1/2_ parameter, and as a function of the constant (and hence known as *bias*) current *I*_*bias*_, applied intra-cellularly, which is another parameter receiving special attention in the relevant literature

The Discussion provides a succinct summary of the most important findings, as well as a generalization of some results to multi-compartmental models.

To facilitate reading and provide for a wider audience, the most technical sections on automaticity and on the codimension-2 bifurcation structure in the Δ*V*_1/2_ × *I*_*bias*_ parameter plane are presented separately as Supplementary Results.

## Methods

### Single-node model

In this work, we explore the nonlinear dynamics for the following 4D system of ordinary differential equations (ODE):

$$\begin{array}{c} C_{m}\frac{dV}{dt}=I_{s}-I_{ion} \end{array}$$ (1)

where *I*_*s*_ stands for the stimulation current injected into the modeled compartment and

$$\begin{array}{c} I_{ion}\equiv g_{Na}m^{3}h\left(V-E_{Na}\right)+g_{K}n\left(V-E_{K}\right)+g_{leak}\left(V-E_{leak}\right) \end{array}$$

is the total ionic current. The channel-gate state variables of the HH model *m*, *h* and *n* are described by:

$$\begin{array}{ccc} \tau_{m}\left(V|\Delta V_{1/2}\right)\frac{dm}{dt} & = & m_{\infty }\left(V|\Delta V_{1/2}\right)-m\\ \tau_{h}\left(V|\Delta V_{1/2}\right)\frac{dh}{dt} & = & h_{\infty }\left(V|\Delta V_{1/2}\right)-h\\ \tau_{n}\left(V\right)\frac{dn}{dt} & = & n_{\infty }\left(V\right)-n \end{array}$$

Matlab was used to numerically solve the model Eq (1) and AUTO [33] —for fixed point (FP) or periodic orbit (PO) continuation and bifurcation analysis.

We use the single-compartment Hodgkin-Huxley model as described in [34]. The same model is also used in [24, 25] as a building block. There we noticed an interesting pattern in the description of the *Na*_*v*1.2_ and *Na*_*v*1.6_ channel subtypes. For *both* the *m* and *h* gating variables, *all* the related *V*_1/2_ parameters differed by exactly 13mV.

From whole-cell expression of the *Na*_*v*1.6_ subtype, *V*_1/2_ = −29.2±1.8 mV and *V*_1/2_ = −29.4±1.6 mV (as reported by [10] and [11] respectively). For the *Na*_*v*1.2_ subtype, *V*_1/2_ = −18.32 mV [29]. We also noticed that the cited modeling and experimentally observed data preserved a quite similar *relative* difference in *V*_1/2_—between *Na*_*v*1.6_ and *Na*_*v*1.2_. Furthermore, [29] demonstrated the remarkable effects of the pentapeptide QYNAD. At 200 *μM* concentration, the *Na*_*v*1.2_ subtype *V*_1/2_ was increased to −9.15 mV—an almost 9 mV *shift* toward decreased neuronal excitability. Since QYNAD (Gln-Tyr-Asn-Ala-Asp) is endogenously present in human cerebrospinal fluid (CSF) [29], such chemical interactions may also occur naturally and thence have significant effects on neural dynamics.

**Fig 1:**
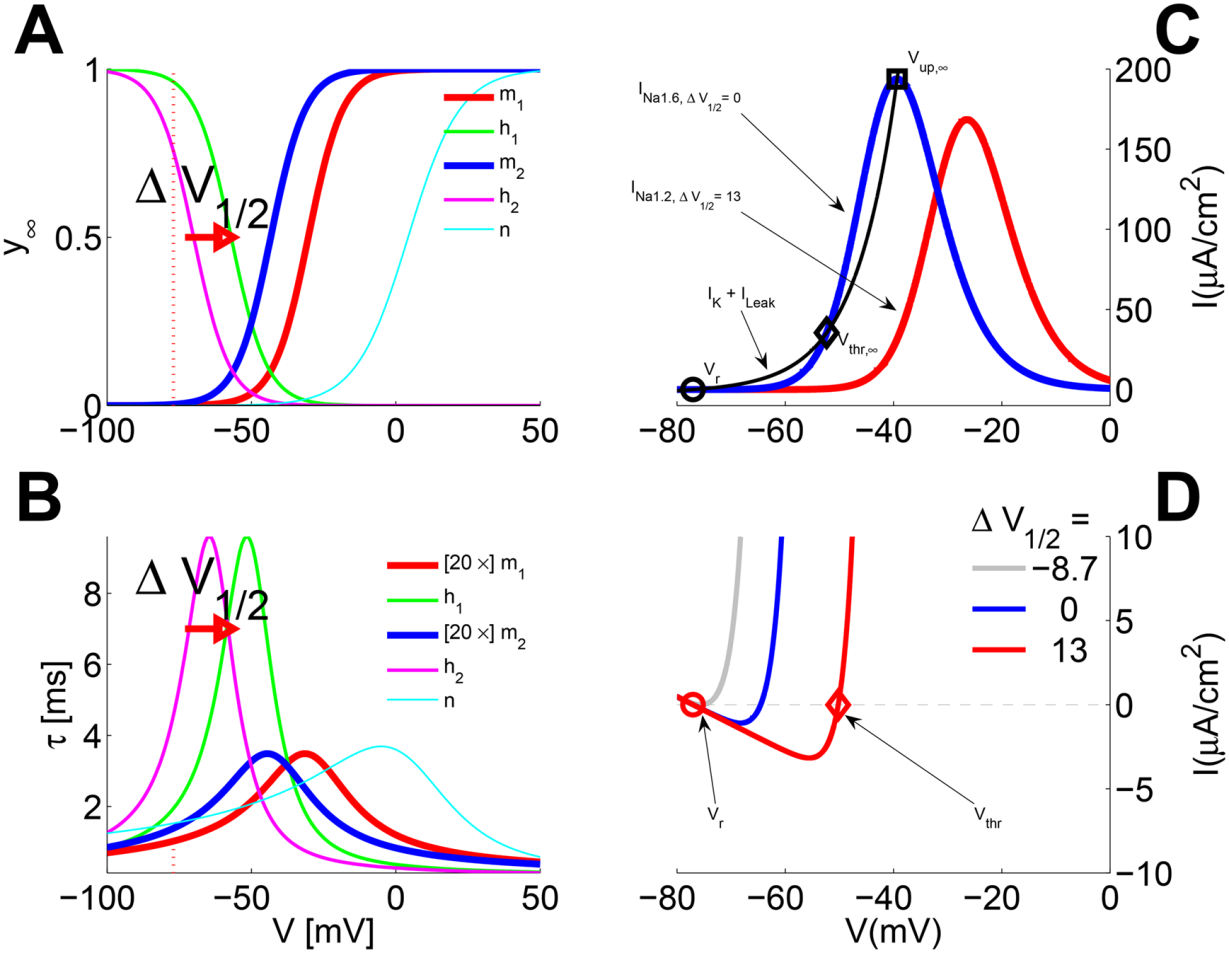
Gate variables as functions of the membrane voltage (*V*). **A:** Asymptotic state *y*_∞_(*V*). **B:** Time constants *τ*_*y*_(*V*) The subscripts 1 and 2 of the *m* and *h* variables refer respectively to the *Na*_*v*1.2_ and *Na*_*v*1.6_ ion channels. Panels C and D illustrate the ionic currents interplay in the model as a function of membrane voltage *V*, computed for different example values of Δ*V*_1/2_. The *color* traces use a color-code compatible to panels A, B for the two key *Na*_*v*_ channel currents; the latter plots are *inverted* to facilitate the interpretation of equilibria. **C:** *I*_*ion*,∞_[Δ*V*_1/2_](*V*), computed for the *asymptotic* state *y*_∞_(*V*) of all gate variables The *thin black* trace shows the total *re-polarization* current *I*_*K*_ + *I*_*Leak*_ Fixed points (FP’s) locations are illustrated through the interplay of *I*_*ion*,∞_(*V*) components **D:** *I*_*ion*,0_[Δ*V*_1/2_](*V*)—computed for *m* = *m*_∞_[Δ*V*_1/2_](*V*) similarly to *I*_*ion*,∞_(*V*), but for the reduced (1D) model at the *resting* state of the slower *h* and *n* gates, i.e. *h* = *h*_*rest*_ = *h*_∞_[Δ*V*_1/2_](*V*_*rest*_) and *n* = *n*_*rest*_ = *n*_∞_(*V*_*rest*_), where *V*_*rest*_ = the resting potential. The circle markers in both Panels C and D indicate the resting-state FP *V*_*rest*_. The diamond markers indicate the second AP-threshold FP *V*_*THR*_, which for *I*_*ion*,∞_(*V*) (panel C) exists only for the *M*3-class models (see text and Fig 4). The gray trace is for Δ*V*_1/2_ = -8.7 mV →*LP*2 (see Fig 4).

The *Na*_*v*1.6_ and *Na*_*v*1.2_ channel types can be seen as two instances of a generalized *Na* ^+^ ionic current model, in which the *V*_1/2_ parameter of *both* the *m* and *h* gate variables is controlled by the *parameter* Δ*V*_1/2_ with values of 0 *mV* and 13 *mV*, respectively (Fig 1 and Tables 2, 3 and 4; see also the gate-state dynamics maths in Box 1.

**Table 2:** The *V*_1/2_ parameter values [mV], by ion-channel type—from [24, 25].

Rate parameter	*Na*_*v*1.6_		*Na*_*v*1.2_		*K*
	*m*	*h*	*m*	*h*	*n*
*α*	-41	-48	-28	-35	25
*β*	-41	-73	-28	-60	25
*h*_∞_	-	-70	-	-57	-

**Table 3:** Gate-state dynamics parameters (see also Box 1—key notes on gate-state dynamics).

Notation	Variable description	Value
Temperature dependence:		
*Q*10	*Q*_10_ constant (*1)	2.3
*K* ^+^: *n*-gate (*2)		
*a*_*n*_	*n*-gate max opening rate	0.02
*b*_*n*_	*n*-gate min closing rate	0.002
*k*_*n*_	*n*-gate membrane-voltage-change constant *k*	9
$Na_{v1.X}^{+}$: *m*-gate (*2)		
*a*_*m*_	*m*-gate max opening rate	0.182
*b*_*m*_	*m*-gate min closing rate	0.124
*k*_*m*_	*m*-gate voltage-change constant *k*	6
$Na_{v1.X}^{+}$: *h*-gate (*2,*3)		
*a*_*h*_	*h*-gate max opening rate	0.024
*b*_*h*_	*h*-gate min closing rate	0.0091
*k*_*h*_	*h*-gate constant *k* (*3) used toward *τ*_*h*_ only	5
*k*_*h*_∞__	constant *k* (*3) for the asymptotic gate-state *h*_∞_ only	6.2

*Notes:*: *1: The temperature-dependence factor. Please see the first note in Box 1. *2: Gate-state dynamics parameters of the *K* ^+^ or $Na_{v1.X}^{+}$ channels. Actual channel opening/closing rates are given by Boltzmann-like functions—for details see the second note in Box 1. *3: The $Na_{v1.X}^{+}$ channels’ inactivating *h* gates have an *asymptotic* state, which is independent of the *h* gates’ opening/closing rates—see the second and third notes in Box 1.

**Table 4:** Definition and notation for the key HH variables.

Notation	Variable description and units	Typical value (*1)
Potentials, in *mV*:		
*V*_*m*_ or *V*	Membrane voltage	(*3)
*V*_*rest*_	Membrane resting voltage	-77
*E*_*K*_	*K* ^+^ Nernst potential	-90
*E*_*Na*_	*Na* ^+^ Nernst potential	60.0
*E*_*Leak*_	Leak reversal potential	-70
Membrane capacitance, in *μF*/*cm* ^2^:		
*C*_*m*_ or *C*	Membrane capacity	
*c*	Membrane capacitance	1
Maximum (*2) conductances, in *mS*/*cm* ^2^:		
*g*_*K*_	*K* ^+^ conductance	150
*g*_*Na*_	*Na* ^+^ conductance	300
*g*_*Leak*_	Leak conductance	0.033
Currents, in *μA*/*cm* ^2^:		
*I*_*K*_	*K* ^+^ Ionic Current (*4)	*g*_*K*_ × *n* × (*V*_*m*_ − *E*_*K*_)
*I*_*Na*_	*Na* ^+^ Ionic Current	*g*_*Na*_ × *m* ^3^ *h* × (*V*_*m*_ − *E*_*Na*_)
*I*_*Leak*_	Leak Current	*g*_*Leak*_ × (*V*_*m*_ − *E*_*Leak*_)

*Notes:* *1: Typical values are for the *Na*_*v*1.6_ model, [25]; see also Table 3 *2: These are dependent on (grow with) temperature, the values listed are for *T* = 23°*C* *3: Membrane voltage is either at its resting value *V*_*rest*_; is *depolarized* (grows due to stimulation and/or activated sodium *Na* ^+^ ion channels); is *repolarized* (decays back to *V*_*rest*_, due to the potassium *K* ^+^ ion channels) *4: Ionic currents depend on both the membrane voltage and the dynamic state of the ion channels’ gates. See Table 3.

**Box 1: Gate-state dynamics mathematical details**

*1: Temperature dependence assumed linear with slope $k_{T}=Q_{10}^{(T-T_{0})/10}$, where *T*_0_ = 23°*C*.

*2: Opening/closing rate of gates *y* in the *K* ^+^ or $Na_{v1.X}^{+}$ channels have Boltzmann-like *templates* (⋅_*y*_ subscripts dropped to simplify notation):

$$\begin{array}{ccc} \alpha (a,w) & = & aw/(1-e^{-w/k})\\ \beta (b,w) & = & -bw/\left(1-e^{w/k}\right)=\alpha_{y}\left(b,-w\right)=bwe^{-w/k}/\left(1-e^{-w/k}\right) \end{array}$$

where *w* = *V*_*m*_ − *V*_1/2_, and the rates change in function of *w*:

$$\begin{array}{c} \alpha^{\prime }\left(w\right)=\frac{a}{1-e^{-w/k}}-\frac{awe^{-w/k}}{k{\left(1-e^{-w/k}\right)}^{2}}\;\beta^{\prime }\left(w\right)=\frac{-b}{1-e^{w/k}}-\frac{bwe^{w/k}}{k{\left(1-e^{w/k}\right)}^{2}} \end{array}$$

When *both* the opening and closing rates share the same *V*_1/2_ parameter (e.g. the *m* and *n* gates, Table 2), the corresponding *V*-dependent *asymptotic* gate state and time constants are:

$$\begin{array}{ccc} y_{\infty }\left(w\right)=\lim_{t\to \infty }y\left(t,w\right) & = & \frac{\alpha }{\alpha +\beta }=\frac{a}{a+be^{-w/k}}\\ \tau (w) & = & \frac{1}{\alpha +\beta }=\frac{1-e^{-w/k}}{w(a+be^{-w/k})} \end{array}$$

*α*′(*w*), *β*′(*w*), and *y*_∞_(*w*) are all sigmoidal functions of *w*. *Regardless* of *a*, *b*, *k* values, rate-change dependence on *w* is most important at *w* = 0, where *α*′(*w*) = *a*/2 and *β*′(*w*) = −*b*/2. Compare to *α*′(*w*) ≈ *β*′(*w*) ≈ 0 for *w* ≫ 0.

$y_{\infty }^{\prime }\left(w\right)$ is reciprocally dependent on *k*, and its maximum is obtained at w* = −*k*ln(*a*/*b*) (hence with *a* = *b*, w* = 0). Hence *dy*_∞_(w*)/*dw* is determined by the *V*_1/2_ parameter, and *y*_∞_(w*) = 1/2—*regardless* of the actual *a*, *b*, *k* values.

$$\begin{array}{c} y_{\infty }^{\prime }\left(w\right)=\frac{abe^{-w/k}}{k{\left(a+be^{-w/k}\right)}^{2}}\;y_{\infty }^{\prime \prime }\left(w\right)=\frac{abe^{-w/k}\left(be^{-w/k}-a\right)}{k^{2}{\left(a+be^{-w/k}\right)}^{3}} \end{array}$$

Also for *V*_*m*_ ≈ *V*_1/2_, *τ*(*V*) = *τ*(*w*) attains its maximum (*τ*(w*) ≈ *τ*(0) = 1/*k*(*a* + *b*)), since:

$$\begin{array}{c} \tau^{\prime }\left(w\right)=\frac{\left[\left(a+b\right)w+k\left(a-b\right)\right]e^{-w/k}+k\left(be^{-2w/k}-a\right)}{kw^{2}{\left(a+be^{-w/k}\right)}^{2}} \end{array}$$

E.g. for *a* = *b* = *k* = 1, the numerator of *τ*′(*w*) reduces to *u* ^2^+2*uw* − 1 (*u* = **e** ^−*w*^), which vanishes when *w* = 0.

*3: Inactivating *h* gates of the $Na_{v1.X}^{+}$ channels have the *asymptotic* state

$$\begin{array}{c} h_{\infty }\left(V\right)=1/\left(1+e^{w_{h}/k_{h_{\infty }}}\right)\;w_{h}=V_{m}-V_{1/2,h_{\infty }} \end{array}$$

### Model extensions

#### A Mixture model

provides a comparative perspective for the effects of parameter variation, and specifically concerning the effects of Δ*V*_1/2_ to those of channel density by types and numbers (i.e. *g*_*Na*_ variation). In this model extension, a fraction *P* ≤ 1 of all the *Na* ^+^ ion channels are of the *Na*_*v*1.6_ type (i.e. Δ*V*_1/2_ = 0 mV), while the remaining *Na* ^+^ channels—i.e. a fraction 1 − *P*, are of a second different type of channel with a chosen Δ*V*_1/2_ ≠ 0. Thus:

$$\begin{array}{ccc} I_{Na,\Sigma }\left[P,\Delta V_{1/2}\right]\left(t\right) & = & \left(1-P\right)I_{Na}\left[\Delta V_{1/2}\right]\left(t\right)+PI_{Na}\left[0\right]\left(t\right) \end{array}$$ (2)

Such mixture models may provide predictions of the electrophysiological properties for neural processes with a particular experimentally observed distribution of voltage-gated sodium channel subtypes (e.g. as in [10, 12, 14, 27, 35]).

#### From a compartment to a cell

The single-node model can be generalized to a multiple-compartment model of an entire axon. In models of higher dimensionality, the spatial distribution (relative proportions) of ionic current types will correspond to better or worse conditions for AP initiation and propagation.

To estimate the effect of Δ*V*_1/2_ on AP propagation velocity (through the *Na*_*v*_ currents), homogeneous cables of 100 identical compartments—each of length 25 *μm* (yielding a total cable length of 2.5 *mm*) were simulated (see also [36] for a detailed description of this type of cable models). The cable was subject to the no-flux boundary condition on one end, and the single compartment on the opposite end was stimulated (*T*_*STIM*_ = 100 *μs* or 1 *ms*). For each value of the Δ*V*_1/2_ parameter, the resting threshold was identified. An AP was reliably evoked and propagated to the very end of the cable. A propagating AP was signalled by small deviations of propagation velocity (range < 30% of mean value) in the section occupying the middle 50% of the total cable length.

To avoid overestimating the required stimulation current thresholds, the total duration of simulation was chosen as *T*_*STIM*_ + 1.5 *ms*. However, this also provided for a little extra variability in the identified *I*_*THR*_, due to phenomena resulting from the cable’s finite length and no-flux boundary conditions. Namely, lower (near-threshold) stimulation current latently reaches and depolarizes compartments down the cable. As a result, when the AP is finally evoked it propagates faster than in a significantly “supra-threshold” case. Hence, to avoid excess variability in the estimated propagation velocity, a safety factor was applied: $I_{STIM}=1.5\times {\hat{I}}_{THR}$.

## Results

Our results demonstrate that the continuous variation of the Δ*V*_1/2_ parameter comes close to being equivalent with recent HHM ‘optimizations’. The dynamics of the parametric family exhibit a rich multitude of properties, which we have grouped in 4 main categories, namely: excitability, efficiency, frequency-encoding range and conduction velocity. The former three are discussed in a single-compartment (space-clamp) context. Conduction velocity and whole-neuron excitability are presented within a multi-compartment model extension.

### Effects of Δ*V*_1/2_ variation on neuronal excitability

Can the parameterized HH models’ exhibit wide variation in their excitability?

It is well known that the HHM’s excitability to brief stimulation pulses is fully dependent on the fast activation of the *m* gates versus a slower inactivation of the *h* and activation of the *n* gates (Fig 1A and 1B). This can be demonstrated by a simplified model in which *m* is assumed to reach instantaneously *m*_∞_(*V*) while *h* and *n* remain at their resting values *h*_*rest*_ and *n*_*rest*_. Upon very brief *I*_*s*_ duration this is a fair approximation of the system’s behavior (see Ch.3 in [37]). Stimulating from rest [*V*_*rest*_, *h*_*rest*_, *n*_*rest*_], the system dynamics is reduced to:

$$\begin{array}{c} C_{m}\frac{dV}{dt}=I_{s}-I_{ion}\left(V,m_{\infty }\left(V\right),h_{rest},n_{rest}\right) \end{array}$$ (3)

For all Δ*V*_1/2_, the above approximate ionic current—denoted *I*_*ion*,0_(*V*)—a function of just *V*, has 3 zero-crossings: *V*_*rest*_, *V*_*thr*_ (see Fig 1D) and *V*_*up*_ (not shown). The LOCAL minimum −*I*_*rh*_ is reached for a *V* value located between *V*_*rest*_ and *V*_*thr*_. While *V*_*rest*_ and *V*_*up*_ remain virtually unchanged for different values of Δ*V*_1/2_, *V*_*thr*_ moves toward *V*_*rest*_ and *I*_*rh*_ diminishes as Δ*V*_1/2_ is decreased. In this 1D approximation, one can see that the membrane will depolarize further toward *V*_*up*_ if at the end of the stimulation *V* > *V*_*thr*_. The latter is possible only if *I*_*s*_ > *I*_*rh*_. Hence lower Δ*V*_1/2_ increases the excitability by reducing both *V*_*thr*_ − *V*_*rest*_ and *I*_*rh*_.

**Fig 2:**
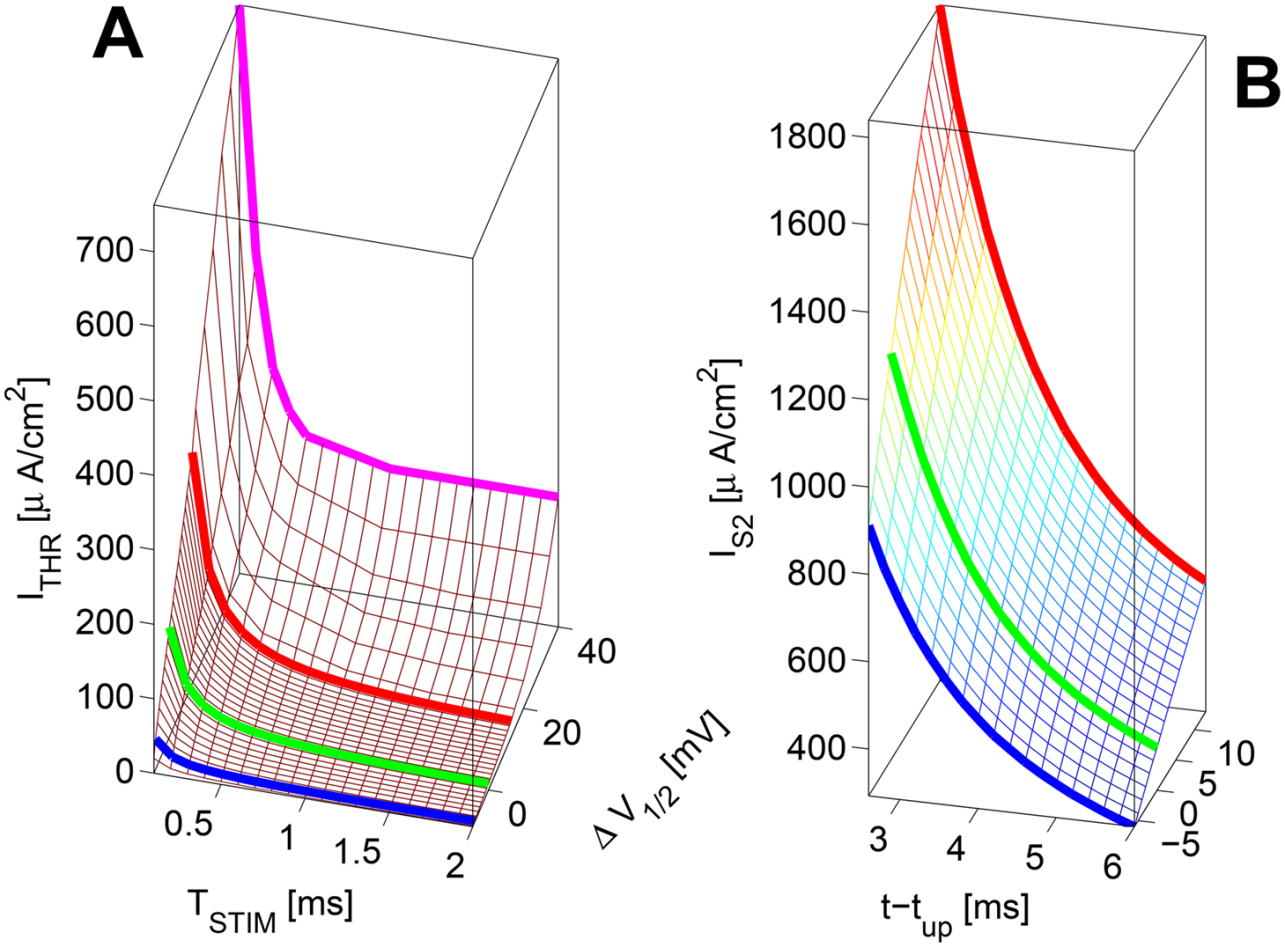
Excitability as a function of Δ*V*_1/2_. **A** Strength/duration (SD) curves; **B** *I*_*S*2,*THR*_(*t* − *t*_*up*_) *T*_*S*1_ = *T*_*S*2_ = 100 *μs*. ∀Δ*V*_1/2_ the test *AP*_*S*1_ is obtained by 1.5× the resting *I*_*THR*_ (see Panel A).

*Strength-duration* (SD) curves for the Δ*V*_1/2_-family, computed through simulations of the full HH dynamics, and for the whole range of Δ*V*_1/2_ ∈ [−8, 40] mV, are shown in Panel A of Fig 2. For the short (e.g. *T*_*STIM*_ = 100 *μs*) the increase of *I*_*THR*_ with Δ*V*_1/2_ is close-to-linear, while for the long (e.g. *T*_*STIM*_ = 2000 *μs*) the increase is steeper than linear.

Toward approximate analytic SD curves, the model is simplified by assuming that all gate variables follow their asymptotic values, i.e.:

$$\begin{array}{c} C_{m}\frac{dV}{dt}=I_{s}-I_{ion}(V,m_{\infty }\left(V\right),h_{\infty }\left(V\right),n_{\infty }\left(V\right)=I_{s}-I_{ion,\infty }\left(V\right) \end{array}$$ (4)

This model is valid for low-amplitude stimulation of very long duration. *I*_*ion*,∞_(*V*) may have one or three zero crossings (Fig 1C). For the one-zero-crossing case, the very existence of a threshold is a *transient* phenomenon, i.e. a finite *V*_*thr*_ no longer exists, when the *h* and *n* gates respectively close and open. With three zero crossings −*I*_*ion*,∞_(*V*) *also* has a local minimum—a situation *qualitatively* similar to the *I*_*ion*,0_(*V*) case above. Hence, just as with the simplified model of Eq (3), further depolarization of *V* toward *V*_*up*,∞_ may be expected when *V* is beyond *V*_*thr*,∞_ at the end of the stimulation. In this case *I*_*rh*_ is *precisely* the value of the rheobase current.

Through *I*_*ion*,∞_(*V*) of Eq (4), a lower bound of the threshold current—the *absolute* rheobase—can be estimated.

Hence, *SD*[*V*_*shift*_] may be estimated for relatively short durations (*T*_*STIM*_ → 0) or long durations (*T*_*STIM*_ → ∞). In the latter case, this is only possible for models like *Na*_*v*1.6_ (Δ*V*_1/2_ = 0), but *not* for models like *Na*_*v*1.2_. This is one of the key properties pointed at in this work.

To evoke an AP, according to Eq (1) the stimulation current *I*_*s*_ needs to overcome the dominating opposing ionic currents *I*_*ion*_. In search for patterns of lowest amplitude or highest energy efficiency, *I*_*s*_ may *just slightly* outweigh *I*_*ion*_ and still successfully trigger AP’s [36]. However, this is highly dependent also on stimulation duration and *Na*_*V*_ class.

Importantly, for the *Na*_*V*1.6_ subtype, but *not* for the *Na*_*V*1.2_ subtype—as demonstrated through the simplified model Eq (4)—a threshold value of the membrane voltage exists for *any* stimulation duration. Hence an AP can be evoked by applying almost as little current as the absolute rheobase current of a sufficient duration (Fig 2A).

Refractoriness (absolute or relative) is the other face of excitability. In a standard procedure to assess it, one applies a second stimulus *S*2 at different times along a test action potential *AP*_*S*1_, induced by an initial supra-threshold stimulus *S*1. The minimal current *I*_*trh*,*S*2_, needed for another active response, is then found for each *S*1 − *S*2 coupling time, or else the system is diagnosed as absolutely refractory—if current amplitude lies beyond some acceptable limit. *I*_*trh*,*S*2_ is a function of *t* = *t*_*S*2_ − *t*_*S*1_ coupling interval (assume *t*_*S*1_ = 0 for simplicity), as well as of the *S*2 duration *T*_*s*,*S*2_. Whether it also depends on the parameters of the *S*1 stimulus is to be investigated. Alternatively, refractoriness can be studied by a one-dimensional approximation similar to Eq (3).

$$\begin{array}{c} C\frac{dV}{dt}=I_{s}-I_{ion}\left(V,m_{\infty }\left(V\right),h_{0},n_{0}\right) \end{array}$$ (5)

in which *h*_0_ = *h*(*V*_0_) and *n*_0_ = *n*(*V*_0_) are the values of the gate variables at each point *V*_0_ along the *AP*_*S*1_.

As for stimulations from the resting state, a necessary (but not sufficient) condition for *S*2 to produce an AP is that Eq (5) has 3 FP’s. When *V*_*thr*_ does not exist, the system is absolutely refractory. Otherwise, a minimum stimulus current is needed.

We consider the case where *AP*_*S*1_ is evoked by a stimulus applied from the resting state. Both *V*_*thr*_(*t*)—calculated from Eq (5), and *I*_*thr*,*S*2_(*t*) depend on the *AP*_*S*1_, which in turn varies with *T*_*s*,*S*1_ (and the corresponding *I*_*s*,*S*1_). However, both functions become invariant once the time axis is aligned to *t*_*up*_—the time of maximum *dV*/*dt* derivative during the upstroke. Different *I*_*s*,*S*1_ and *T*_*s*,*S*1_ may modify the *latency* of *AP*_*S*1_, but almost none of its time course beyond the upstroke. This invariance is maintained as long as *T*_*s*,*S*1_ does not become too long. Conversely, when *T*_*s*,*S*1_ → ∞, *I*_*s*,*S*1_ becomes what is known as *bias* current. It may induce automatic firing, which in turn precludes the use of the *S*1/*S*2 protocol (see also the Supplementary Results subsection on automaticity).

The invariance of the *AP*_*S*1_ time course means that a single *I*_*thr*,*S*2_(*t* − *t*_*up*_) curve (excitability is a function on the *recovery time* *t* − *t*_*up*_) can be used to capture the system behavior for each *T*_*s*,*S*2_ and Δ*V*_1/2_. Rabinovitch et al. [38] referred to this invariant trajectory as *hidden structure* (HS) and proposed a method that extends to excitable systems the concept of the *phase transition curve* (PTC), widely used to analyze the forcing of non-linear oscillators [39, 40].

Panel B of Fig 2 shows the variation of *I*_*thr*,*S*2_ as a function of *t* − *t*_*up*_ and Δ*V*_1/2_ for *T*_*STIM*_ = 0.1 ms. Decreasing Δ*V*_1/2_ slightly shortens the absolute refractory period. For any given *t* − *t*_*up*_, it also reduces the current needed to get a response. For any *t* − *t*_*up*_, there is a quasi-linear increase of the *I*_*thr*,*S*2_ as a function of Δ*V*_1/2_. For *t* − *t*_*up*_ > ≈ 2.5 ms, where excitability regained in all model *Na*_*V*_ subtype cases, a higher Δ*V*_1/2_ may mean up to a two-fold increase of the threshold current.

### The metabolic efficiency of the HHM[Δ*V*_1/2_] family

Can the parameterized HH models exhibit higher metabolic efficiency?

The *Na* ^+^ ions that enter the cell during an action potential (AP) will have to be returned back to the extra-cellular space. This is done by the *Na* ^+^/*K* ^+^-ATPase enzyme pump, while simultaneously bringing the repolarizing-phase *K* ^+^ ions back to maintain the resting potential. It binds 3 intracellular *Na* ^+^ and 2 extracellular *K* ^+^ ions. One ATP molecule is hydrolyzed, leading to phosphorylation and a conformational change in the pump, which exposes the respective ions to alternate sides of the cell’s membrane. Thus the *Na* ^+^/*K* ^+^ pump can be responsible for up to 70% of the neurons’ energy expenditure.

Hence, the metabolic cost for firing a single AP is proportional to the *Na* ^+^ ionic charge that enters the cell during each AP, and also depends on the overlap of opposing *Na* ^+^/*K* ^+^ ionic currents [41].

**Fig 3:**
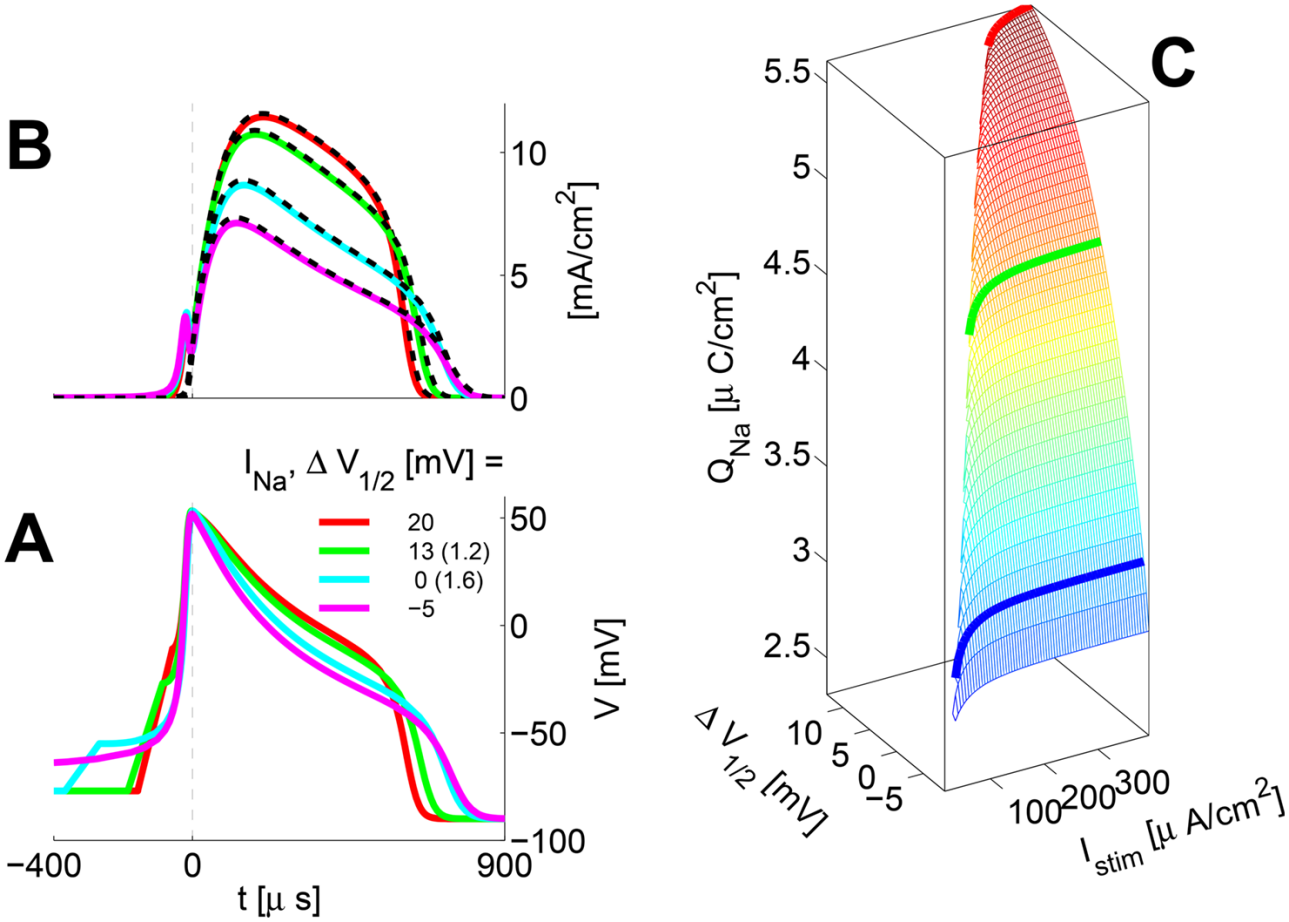
AP properties and Metabolic efficiency as a function of Δ*V*_1/2_. **A** Membrane voltage during the AP; **B** *I*_*Na*_ / *I*_*K*_ interplay; **C** Overall Metabolic efficiency Thick color: −*I*_*Na*_, aligned to the AP upstroke (time zero); Dashed black: the respective *I*_*K*_ *Q*_*Na*_—charge transferred into the modeled compartment during a single AP. AP shape and *Q*_*Na*_ nearly invariant with *I*_*STIM*_ ≫ *I*_*THR*_.

Such AP properties as the *I*_*Na*_/*I*_*K*_ currents interplay and metabolic efficiency as a function of Δ*V*_1/2_ are illustrated in Fig 3. ∀Δ*V*_1/2_ the *AP* is obtained via 1.5× the resting *I*_*THR*_ for duration *T*_*STIM*_ = 100 *μs*. As observed, larger Δ*V*_1/2_ values translate into lower excitability: higher threshold potential *V*_*THR*_ and threshold stimulation currents *I*_*THR*_. The very large latencies for Δ*V*_1/2_ = 0 and -5 *mV* are due to very low threshold values *I*_*THR*_, which remain low even after a 50% “safety-factor” increase. The thick color traces in Panel A of the figure show the −*I*_*Na*_ current profiles, aligned to the AP upstroke (time zero). The *Na* ^+^ current has a local peak *just before* the AP upstroke. there is a complete overlap of the *Na* ^+^ and *K* ^+^ currents (shown by color and dashed-black traces respectively in Fig 3B). The close *I*_*Na*_/*I*_*K*_ interplay here gives an electrophysiological and physical perspective onto the nonlinear dynamics concept of the “slow manifold” [37, 42, 43].

As Fohlmeister and colleagues have observed [7, 35], in the *classical* HH model the large overlapping *Na* ^+^ and *K* ^+^ currents lead to considerable (and rather ‘suboptimal’) metabolic energy expenditure.

However, smaller Δ*V*_1/2_ values reduce *both* the *Na* ^+^ and *K* ^+^ currents through the AP’s re-polarization stage. First, lower Δ*V*_1/2_ implies lower *V*_*THR*_. Second, the AP upstroke amplitude *V*_*up*_ is invariant to Δ*V*_1/2_ and hence *h*_∞_(*V*_*up*_) is significantly more closed for smaller Δ*V*_1/2_ values (see Fig 1A).

The resting FP *V*_*rest*_ remains almost identical in the *Na*_*v*1.2_ and *Na*_*v*1.6_ models (-77.03 vs -77.01 mV), but nevertheless corresponds to quite different values of *h*_∞_(*V*_*rest*_) (0.96 vs 0.76). Hence, the *Na*_*v*1.6_ h gates will close more during the AP upstroke, and will recover later and to a lesser value during the post-upstroke re-polarization. Furthermore, the *Na*_*v*1.6_ model has two additional depolarized FP (diamond and square in Fig 1C—denoted *V*_*thr*,∞_ and *V*_*up*,∞_).

**Fig 4:**
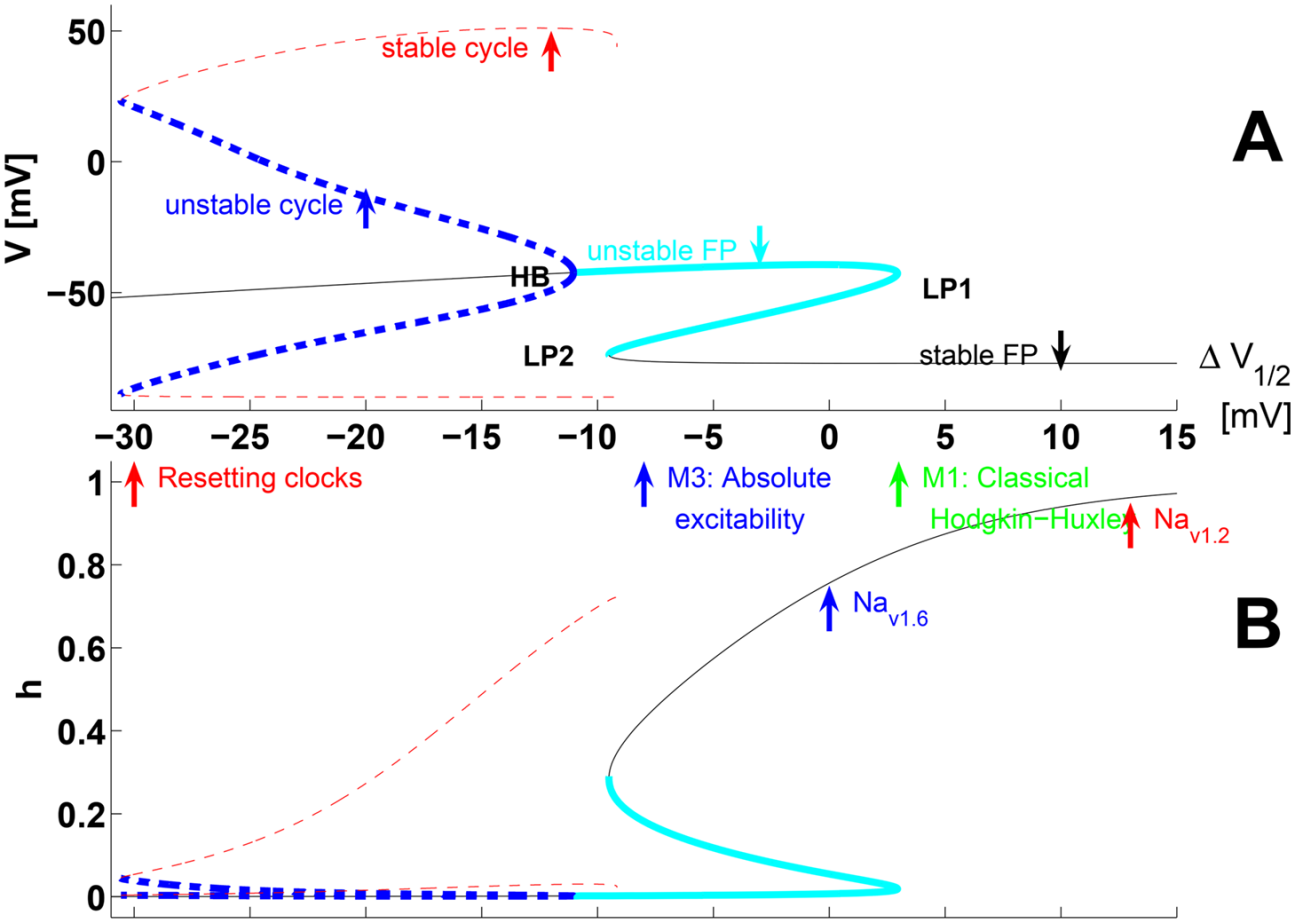
State-space dynamics and model regimes as a function of Δ*V*_1/2_. These bifurcation diagrams illustrate the FP’s and PO’s of the *V* and *h* model states as a function of Δ*V*_1/2_. *Thin black line*: stable FP’s; *thick cyan*: the unstable-FP branches (with one or two (real or complex) positive-real eigenvalues. *LP*1 and *LP*2: lower and upper limits of the middle branch. HB: Hopf bifurcation. See the text for more details. Thin and thick dashed lines: minimum and maximum model-state value for the stable and unstable cycles respectively. Unstable cycles are born from FP’s at a *subcritical* HB and metamorphose to stable at a cycle saddle-node (fold).

Compare the equilibria of the gate state *h* on Fig 4 for the typical models of *Na*_*v*1.6_ (Δ*V*_1/2_ = 0), and *Na*_*v*1.2_ (Δ*V*_1/2_ = 13). The latter figure contains the big picture of our bifurcation analysis (presented next in more detail), namely that the Δ*V*_1/2_ family contains as a superset a wide variety of models with different properties, in which models behaving exactly like the *classical* HHM archetype are encountered over only a sub-interval of the Δ*V*_1/2_ parameter full range of variation.

Efficiency is estimated by computing the overall *Na* ^+^ ions’ charge *Q*_*Na*_ (in micro-Coulomb’s per *cm* ^2^), which is transferred during a single AP ([41, 44], *T*_*STIM*_ = 100 *μs*). The integration is carried over the whole time span (30 *ms*) of the simulation (from AP triggering, incl. latency to the return to rest). The shape of the AP and hence *Q*_*Na*_ are mostly invariant as long as *I*_*STIM*_ ≫ *I*_*THR*_. A lower Δ*V*_1/2_ clearly associates with a lower estimate of metabolic cost (see Fig 3, Panel C).

Finally, is this effect trivial? Can it be easily deduced by looking at the HHM Eq (1) alone? Here we argue that this is not the case:

Without a significant effect on AP amplitude or duration (Fig 3A and 3B), the overall shape of the AP changes just slightly and a *significant* gain in metabolic energy expenditure is achieved by an earlier *closing* of the voltage-gated *Na* ^+^ channels. Since *V*_*THR*_ is lower in this case, this earlier *Na* ^+^ inactivation is *matched* to a significantly lesser activation of the voltage-gated *K* ^+^ channels.

### Bifurcation structure as a function of the Δ*V*_1/2_ parameter

The resting potential (*V*_*rest*_) of the system is a *fixed point* (FP) of the system set by:

$$\begin{array}{c} I_{ion,\infty }\left(V\right)=G_{Na}m_{\infty }{\left(V\right)}^{3}h_{\infty }\left(V\right)\left(V-E_{Na}\right)+G_{k}n_{\infty }\left(V\right)\left(V-E_{k}\right)+G_{leak}\left(V-E_{leak}\right)=0 \end{array}$$ (6)

It is located at the intersection of the monotonically increasing outward currents *I*_*K*,∞_(*V*) + *I*_*leak*_(*V*) with the bell-shaped Δ*V*_1/2_-dependent inward current *I*_*Na*,∞_ (Fig 1C). The effect of Δ*V*_1/2_ variation is a linear *shift* of the window-current *I*_*Na*,∞_[Δ*V*_1/2_](*V*) toward lower or higher *V* values. This may create additional FP’s. The number, location and stability of the FP’s is bound to play a significant role in the dynamic properties of the system upon stimulation and re-polarization.

**Fig 5:**
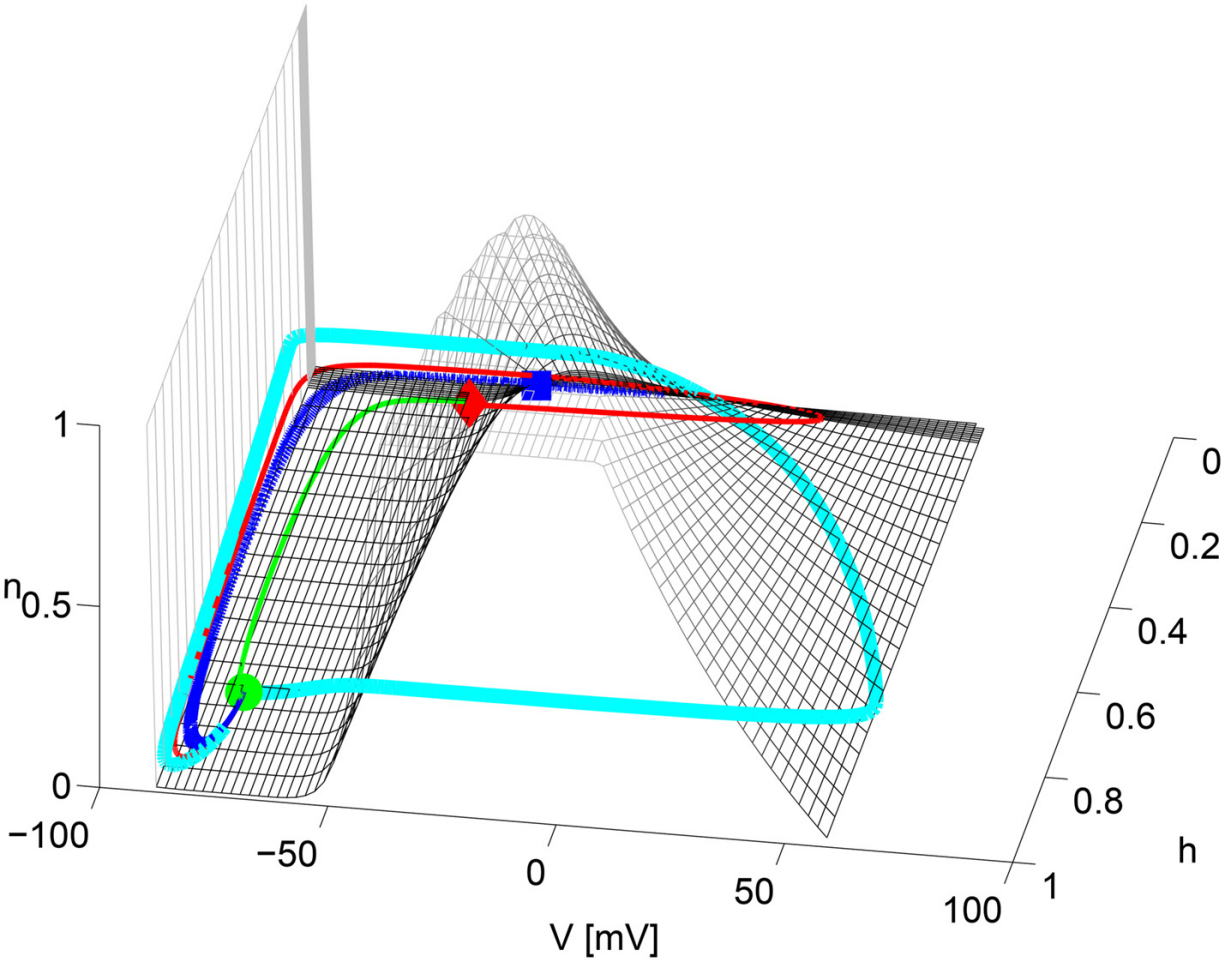
Complex dynamic organization for Δ*V*_1/2_ = 0 *mV*. Two heteroclinic trajectories, starting at *V*_*THR*_ (**red diamond**) and ending at *V*_*rest*_ (**green circle**): correspond to passive—**green** and active depolarization—**red**. Trajectories from *V*_*UP*_ (**blue square**)—thin **dotted blue** connect into *V*_*r*_. All trajectories avoid and “slide” on the *slow* manifold (SM) (**wireframe** mesh), constructed by solving *I*_*ion*_(*V*, *m*_∞_(*V*), *h*, *n*) = 0 ∀(*n*, *h*). Its ‘cubic-like’ shape defines the excitation threshold.

The existence of the two unstable fixed points also changes the organization of the trajectories in the phase plane. As mentioned in the presentation of the bifurcation diagram below, *V*_*thr*,∞_ has a single unstable direction. Perturbations along this direction (either positive or negative) produce two heteroclinic trajectories ending at *V*_*rest*_, corresponding to a passive and an active depolarization (Fig 5, the red and green trajectories). The latter is the limiting case of an action potential that would be obtained by a stimulus slightly beyond the rheobase current. Another set of trajectories connect the most depolarized fixed point (unstable focus) *V*_*up*,∞_ to the stable resting state. All these invariant trajectories and their associated stable manifold do not exist for models like that of the *Na*_*v*1.2_ subtype. They add constraints onto the system dynamics since they cannot be crossed by any other trajectory. As we shall see below, the two additional FP’s also affect the nature of the automatic regimes upon sustained stimulation.

Fig 4A shows the bifurcation structure diagram (BD) of the of the 4*D* space-clamp model as a function of the Δ*V*_1/2_ parameter. In addition to the resting point (the lower branch on the BD, see the “stable FP” arrow), a second depolarized FP (corresponding to *V*_*thr*_ in panel A) appears through a saddle node bifurcation at the limit point **LP1**: Δ*V*_1/2_ = 2.94 mV. From **LP2**: Δ*V*_1/2_ = -9.5 mV, up to **LP1**, the *S*-shaped BD FP curve has three coexisting branches. Beyond **LP2**, a single depolarized FP remains. FP stability is also accounted for in Fig 4A. The middle branch (**LP1** to **LP2**) is *half-stable* (see Table 5 and [43]) due to a single positive real eigenvalue. The lower FP branch is stable with four real negative eigenvalues, except for a tiny interval close to **LP2** (there is a second subcritical Hopf bifurcation **HB2** located 0.005 *mV* beyond LP2, not shown). The uppermost branch is unstable from **LP1** down to the Hopf bifurcation point (**HB**: Δ*V*_1/2_ = -11.05 mV), where it recovers stability. From **LP1** to **HB** the top branch has either two positive real (Δ*V*_1/2_ ∈ [1.7 mV, **LP1**]), or two positive-real complex eigenvalues (Δ*V*_1/2_ ∈ [HB, 1.7 mV]).

**Table 5:** Bifurcation-terms Glossary [43, 49].

Term	Description
FP stability	A given FP is stable if *all* the eigenvalues of the associated Jacobian have non-negative real parts; an FP is called *half-stable* (e.g. in [43]) when the above condition is not met for *some* of the eigenvalues - in that case the associated eigen-directions diverge from the FP
*Codimension 1 bifurcations*	
Saddle-node, or fold, or limit point (LP)	Two fixed points appear or disappear coalescing onto a single *half-stable* FP—having stable, unstable and neutral (zero-real-part) eigenvalues, which in its turn disappears
Transcritical bifurcation	A given FP may change stability with parameters variation standard mechanism, related to the SN
Hopf bifurcation (HB) *Supercritical* *Subcritical*	a given PO’s amplitude decreases until the PO is reduced to a point and disappears; the PO’s period approaches a *finite* limit (given by the purely imaginary eigenvalues) for the critical parameter value the HB-related PO appears at the HB and its amplitude increases gradually, coexisting with the former FP which has become unstable. within a parameter range the HB-related PO’s—a stable and an unstable of lower-amplitude, coexist with a stable FP, the parameter-dependent system trajectory *jumps* to a distant attractor, and hysteresis-like phenomena occur as the parameter is either increased or decreased.
Saddle loop or *homoclinic*	a given PO’s amplitude increases until it captures a saddle point and disappears; the PO’s period a →∞ as the parameter approaches the critical value
Double cycle or saddle-node of cycles (SNC)	Two POs appear or disappear coalescing onto a single *half-stable* PO, which in its turn disappears
*Codimension 2 bifurcations*	
Cusp (C)	Three fixed points appear or disappear this is a sort of LP generalized by the presence of the second parameter
Takens-Bogdanov (TB)	An LP and HB coalesce into an FP yielding an ODE Jacobian with two zero eigenvalues; the HB-related PO becomes a homoclinic.
Generalized Hopf (GHB)	Two HB coalesce, as the second parameter reaches a critical value

The **HB** is *subcritical* (see Table 5) and produces a branch of unstable PO’s connecting through a saddle-node of cycles (SNC) bifurcation to a high-amplitude stable PO at Δ*V*_1/2_ = -35.5 mV. This stable PO branch disappears slightly beyond **LP2** (around Δ*V*_1/2_ = -9.16 mV) through a second SNC bifurcation (see Table 5, as explained in the Supplementary Results—subsection on automaticity). In this work, the analysis is mostly restricted to Δ*V*_1/2_> **LP2**. There are two distinct model sub-classes by the parameter value range:

M1 Δ*V*_1/2_ > **LP1**. The corresponding dynamic system has a single stable resting (hyperpolarized) FP, e.g. the *Na*_*v*1.2_ channel subtype.
M3 Δ*V*_1/2_ ∈ [**LP2**, **LP1**] yields 1 stable resting FP, and two unstable FP’s, e.g. the *Na*_*v*1.6_ channel subtype. As can be inferred by Fig 1C, increase or decrease of the nominal conductivity *g*_*Na*_ offers an alternative way to create or suppress the folding and hence the appearance of the middle and topmost FP branches. Likewise, a variation of the relative proportion of different channel subtypes in a mixture model is equivalent to nominal conductivity variations. Hence, an estimate of the *g*_*Na*_ value yielding a transition from one to 3 FP’s is useful to understand the dynamics of the mixed model. From Fig 1C, and with Δ*V*_1/2_ = 0 mV, *g*_*Na*_ has to be decreased by a third to suppress the BD from folding, but with Δ*V*_1/2_ = 13 mV, it has to be increased *fourfold* to create the fold.

Except for a small interval of Δ*V*_1/2_ ∈ [**HB2**, -9.16 mV] where a stable FP and a stable PO coexist, the two model sub-classes necessarily return to their resting state after a sub-theshold stimulus. However, the supra-threshold dynamics (leading to an AP) will be dramatically influenced by the existence or not of the 2 additional FP’s (see the Supplementary Results’ subsection on automaticity).

### Lower Δ*V*_1/2_ brings about a large frequency-encoding range

Can the parameterized classical HH model exhibit the low-frequency automatic firing needed to account for certain regimes observed experimentally in mammalian species?

Before we answer this question, let us introduce another preliminary bifurcation analysis result. It is known [37], that in the HH model periodic firing may be obtained by injecting a constant *I*_*bias*_—known as *bias* current within a certain range (see also Fig A in S1 File). In the literature one can frequently encounter bifurcation analysis of the HHM with respect to *I*_*bias*_.

**Fig 6:**
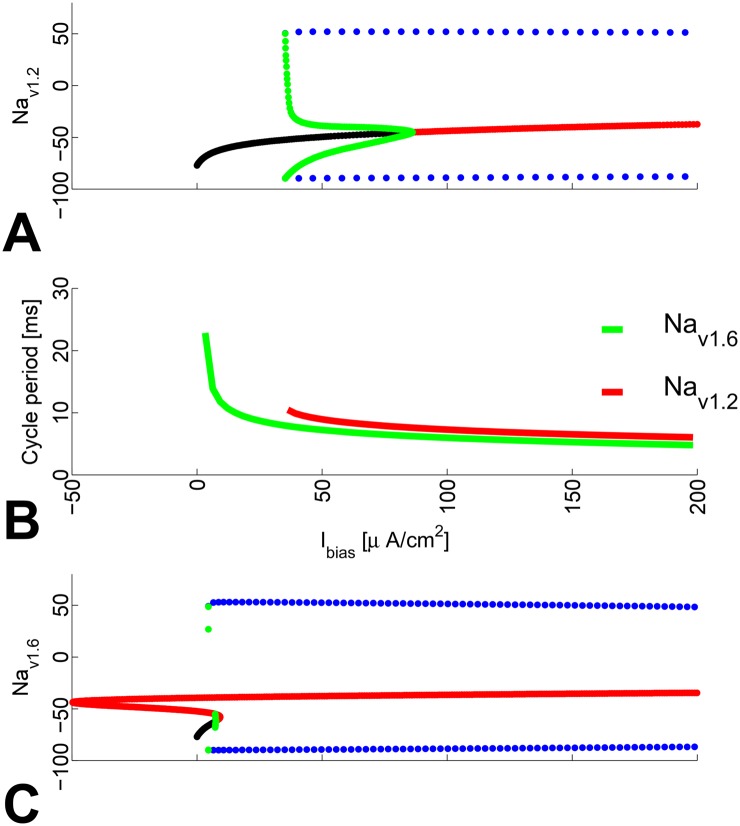
*I*_*bias*_ BD’s for the *Na*_*v*1.2_ and *Na*_*v*1.6_ ion channels (Δ*V*_1/2_ = 13 and 0 mV, resp.).

Panels A and C in Fig 6 result from exactly such bifurcation analysis for the typical models of *Na*_*v*1.6_ (Δ*V*_1/2_ = 0), and *Na*_*v*1.2_ (Δ*V*_1/2_ = 13). Here a higher Δ*V*_1/2_ translates into incapacity for low-frequency automatic firing and a narrow overall frequency-encoding range (see Panel B in Fig 4), consistently with earlier observations [7].

Panel B in Fig 6 illustrates the period length of automatic firing as a function of *I*_*bias*_. In the *Na*_*v*1.6_ case (see also Panel C), stable automatic firing (blue dots in Fig 6B) is lost through a cycle saddle node, which accounts for the finitely increased length of the related cycle period, which is a desirable property of the parametric model family. On the other hand, the unstable cycle (green dots in Fig 6B) disappears into an homoclinic bifurcation, when *I*_*bias*_ tends to the forementioned *absolute* rheobase value. As is well known, homoclinic bifurcations may be associated with infinite increase of the related period.

**Fig 7:**
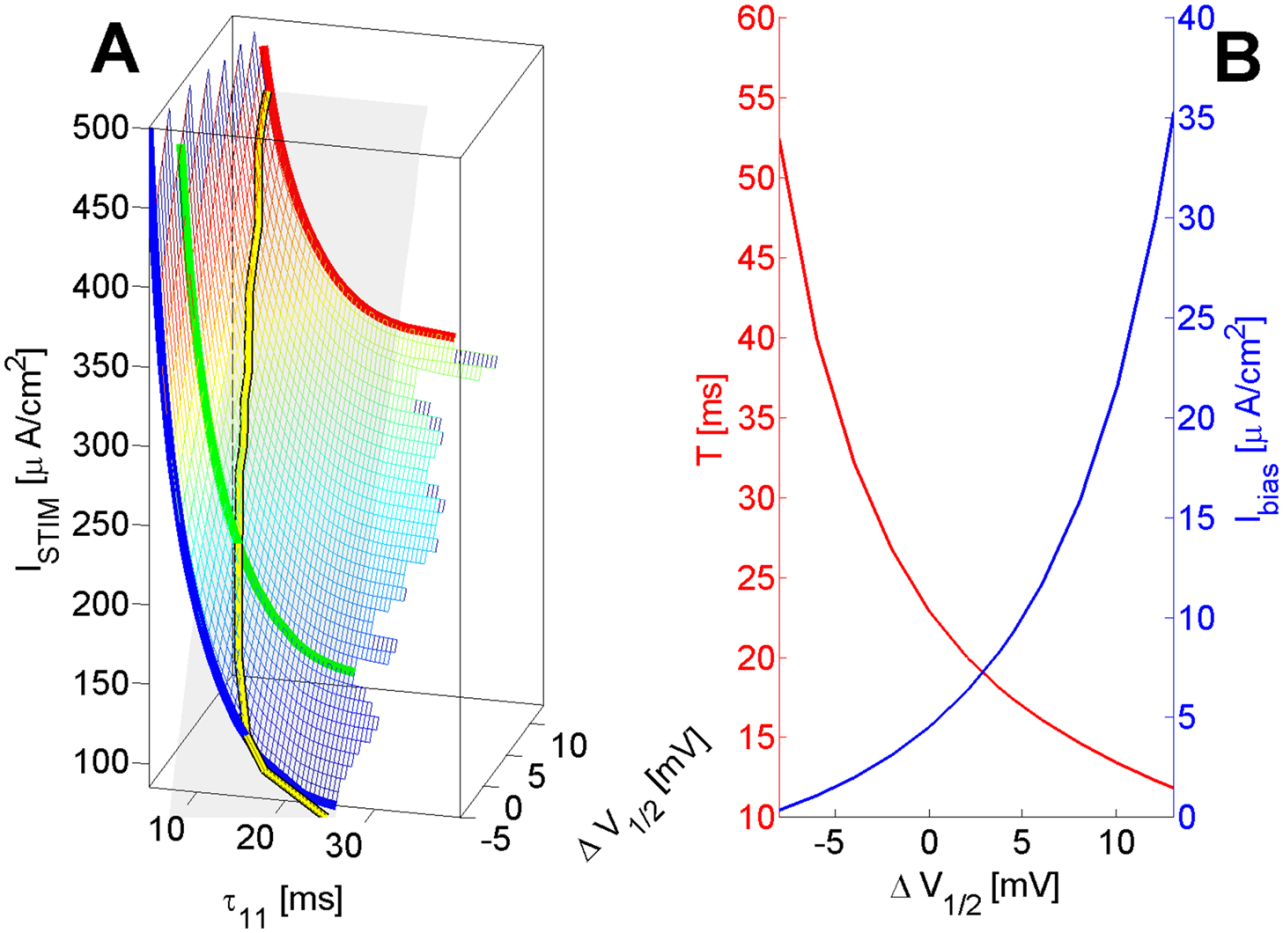
Limits of automatic firing and 1:1 pacing as a function of Δ*V*_1/2_. **A** Stimulation current (also function of frequency); **B** Min. *I*_*bias*_ (→ Max. period length) for stable cycles *I*_*STIM*_ required for an 1:1 response at pacing period *τ*_11_. *T*_*STIM*_ = 100 *μs*; *I*_*STIM*_ → *I*_*THR*_ as *τ*_11_ ≫ 0; Semi-transparent quasi-planar surface = 1.5× the resting *I*_*THR*_.

Fig 7 illustrates the limits of automatic firing and of periodic stimulation maintaining the 1:1 response pattern (also known as *pacing*) as a function of Δ*V*_1/2_. In Panel A of the figure, the *periodically-applied* stimulation current *I*_*STIM*_ required for an 1:1 response—at the given minimal pacing period *τ*_11_, depends on pacing frequency. Clearly, *I*_*STIM*_ → *I*_*THR*_ (the *resting* threshold, see also Fig 2), as *τ*_11_ → ∞. Notice that, with near-threshold current values, higher Δ*V*_1/2_ allow periodic stimulation at higher frequency.

However, the situation is reversed for automatic regimes. The period of automatic firing is a monotonously decreasing function of *I*_*bias*_ with a minimum well below 5 ms. This means that *some* (less excitable) models may not have automatic firing at a frequencies below 200Hz. Importantly, this is a monotonicity property specific to the model at hand, contrasting it to other models (e.g. the Fitzhugh-Nagumo model)—(see Fig 7B—itself, a simplified presentation of Fig D.B in S1 File. The full detail of periodic firing variability and bifurcation structure as a function of Δ*V*_1/2_ and *I*_*bias*_ are provided as Supplementary Results).

How about the maximum period that can be achieved?

Applying the same monotonicity property for the period, it would be longest for the lowest *I*_*bias*_ for which automatic firing is still possible. Lower Δ*V*_1/2_ provide for such regimes with progressively lower firing frequency (longer inter-AP periods). As seen in Panel B of Fig 7, a very reasonable Δ*V*_1/2_ parameter variation translates into a *five-fold* variation of period lengths. Thus, the model family is capable of handling automatic firing at a frequency less than 20Hz.

Hence lower-Δ*V*_1/2_ models bring about a significantly larger frequency-coding range, which in addition requires less metabolic resources.

### On Hogkin’s maximum-velocity hypothesis

Can the parameterized HH models conduct AP’s faster?

**Fig 8:**
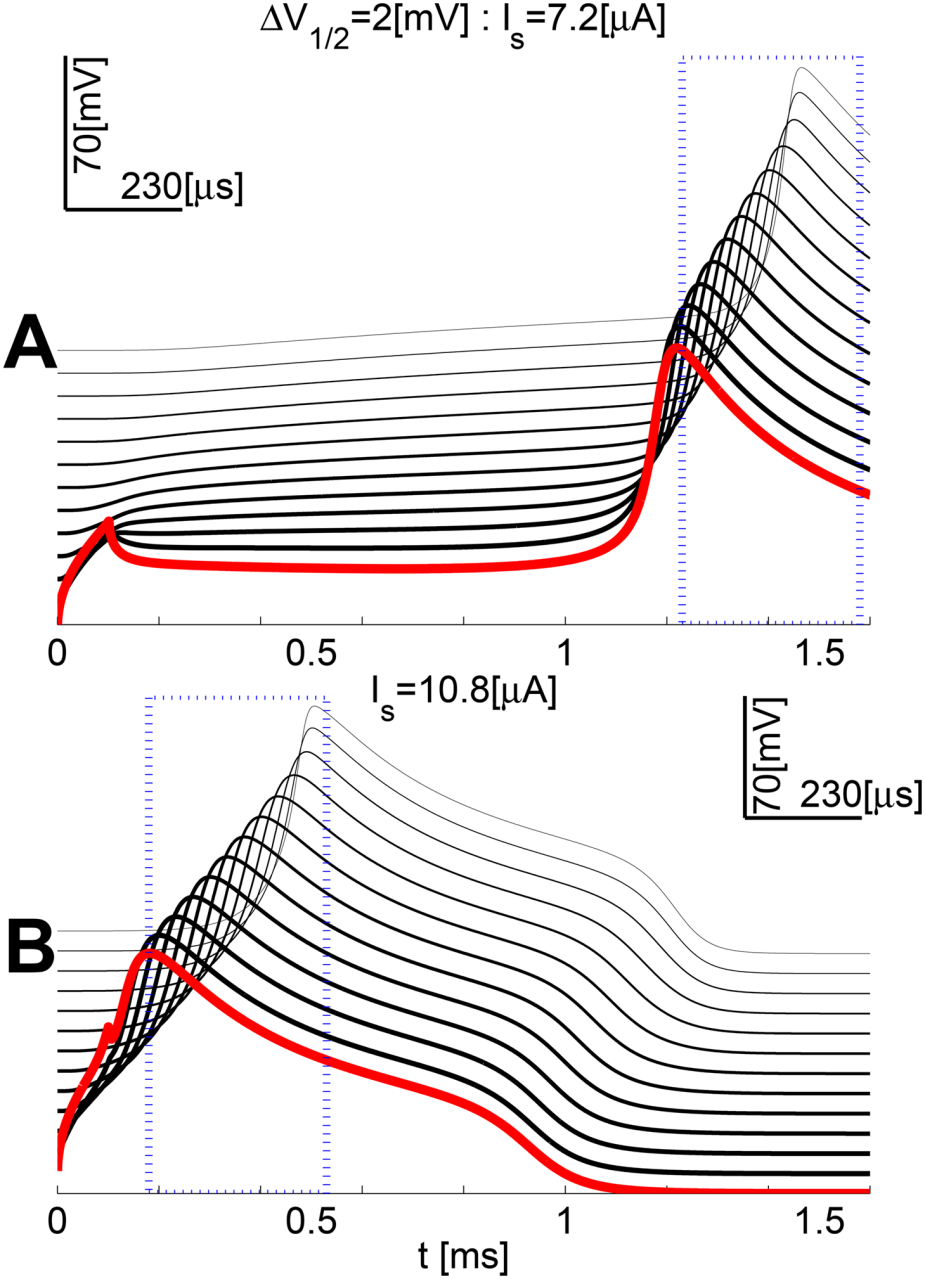
A generalization perspective of Δ*V*_1/2_ variation effects—from a compartment to a cell. **A** When *I*_*STIM*_ is lower, it latently reaches and depolarizes compartments down the cable. This is clearly visible from the voltage traces, which are depolarized up to halfway down. As a result, when the AP is finally evoked it propagates faster. **B** “supra-threshold” case with a safety factor applied $I_{STIM}=1.5\times {\hat{I}}_{THR}$.

The effect of Δ*V*_1/2_ on AP propagation velocity (through the *Na*_*v*_ currents), was studied in a multiple-compartment model as described in the Methods. To reliably evoke propagating AP’s for all values of the Δ*V*_1/2_ parameter, the resting stimulation-current thresholds *I*_*THR*_ were identified. Fig 8 illustrates this for two *post hoc* runs of the central step of the *I*_*THR*_-search algorithm (Δ*V*_1/2_ = 2 mV, please see Methods for a detailed interpretation of the obtained voltage-distribution patterns in either case).

According to Hogkin’s maximum-velocity hypothesis, the ion channels’ ion-types and density evolved through natural selection to maximize the AP conduction velocity. However, it has been argued that gating current limits the increase of *Na* ^+^ channel density as a means to gain in conduction velocity. Moreover, the computed optimal density is more than twice higher than in the real squid, and with capacitance reduced by neglecting gating current, optimal density is even higher [41].

**Fig 9:**
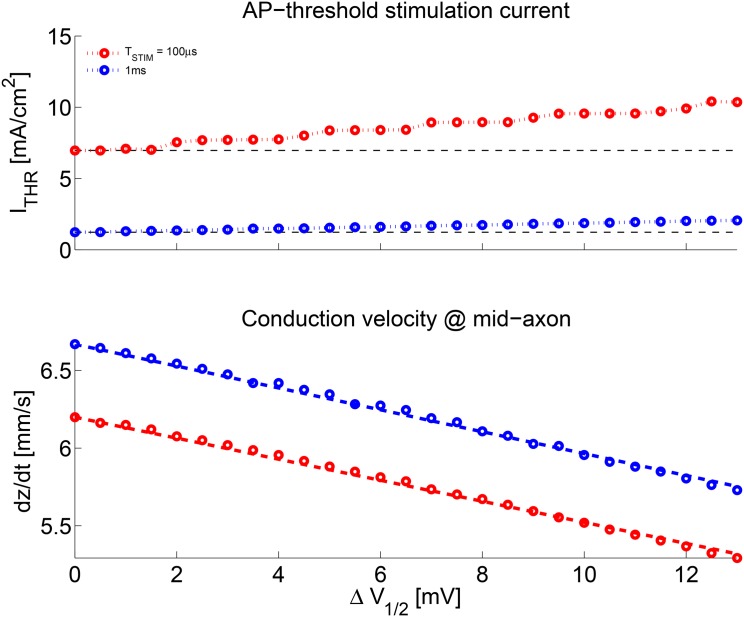
Resting threshold current *I*_*THR*_ and mid-axon mean AP conduction velocity. Notice the clear linear trend of *I*_*THR*_ increasing with Δ*V*_1/2_ for both *T*_*STIM*_ cases. A significant trend of accelerating AP propagation with Δ*V*_1/2_ can be acknowledged. To prevent excessive results variability (see Fig 8 and text), conduction velocity was computed for *I*_*STIM*_ = 1.5 × *I*_*THR*_.

A summary of resting threshold *I*_*THR*_ and mid-axon mean AP conduction velocity as a function of Δ*V*_1/2_ is presented in Fig 9. Notice the clear linear trend of increasing *I*_*THR*_ with Δ*V*_1/2_ for both *T*_*STIM*_ cases. Despite the large estimation variability (as illustrated and discussed in Fig 8) a *significant* trend of deccelerating AP propagation with Δ*V*_1/2_ can be acknowledged.

Hence, evolution may play another card to solve many a problem at once—avoid the overhead of having too many channels, while simultaneously gaining in AP transmission speed, and even in efficiency (as just shown above). All of it by a simple *shift* of the sodium current window along the membrane potential dimension.

### Mixture model

From the preceding analysis, the single-channel model has 3 FP’s for Δ*V*_1/2_ < **LP1** (Fig 4A). This section analyzes mixed models with sodium current. For ease of reference Eq (2) is recalled from the Methods section:

$$\begin{array}{c} I_{Na,\Sigma }\left[P,\Delta V_{1/2}\right]\left(t\right)=\left(1-P\right)I_{Na}\left[\Delta V_{1/2}\right]\left(t\right)+PI_{Na}\left[0\right]\left(t\right) \end{array}$$ (7)

**Fig 10:**
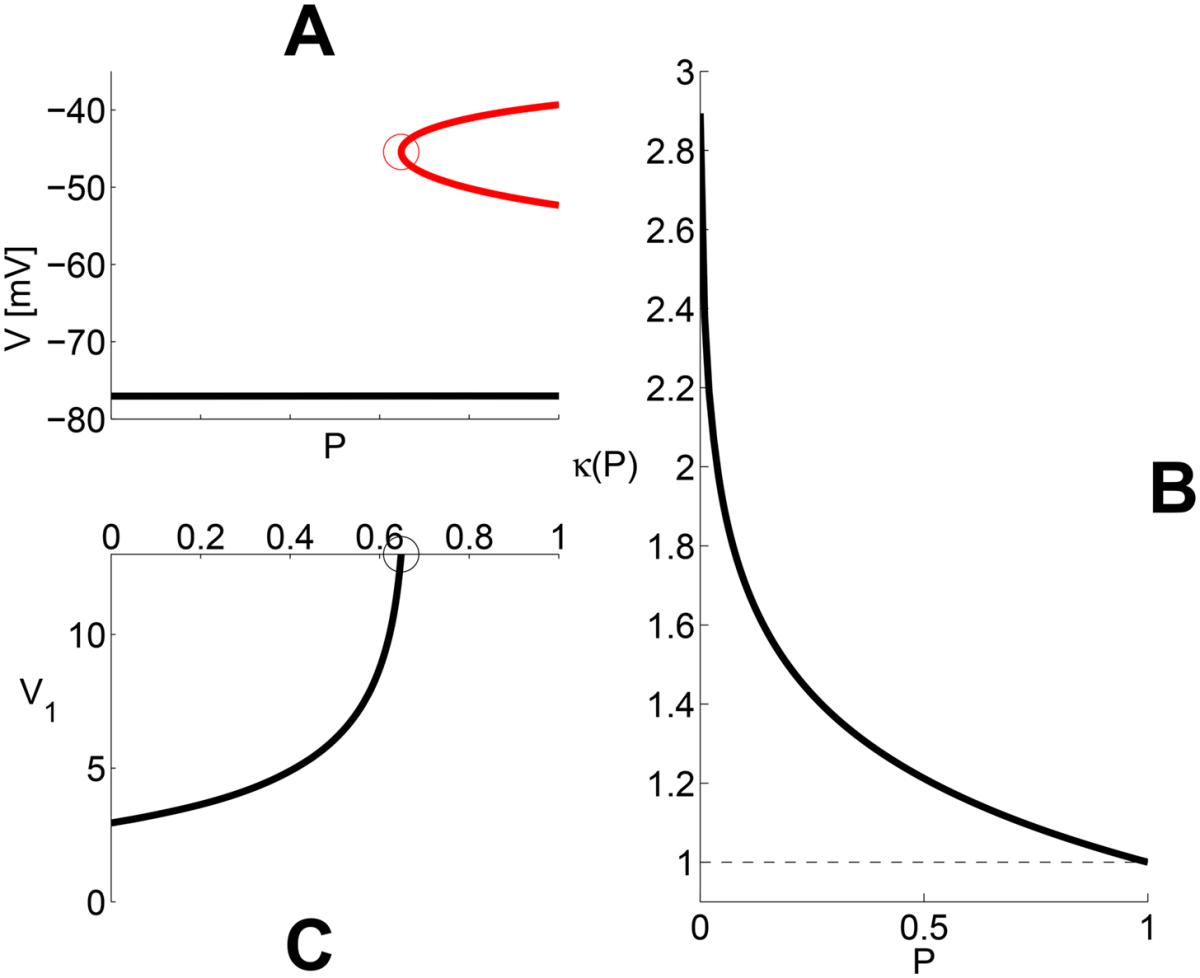
Mixture models. **A:** Red trace: Existence/creation *P* parameter-range for the 2 additional fixed points (saddle-node in the middle branch, and unstable center/focus in the top branch of the B.D.’s) With Δ*V*_1/2_ = 13 mV, *P* ≥ 0.648, which is very close to the minimum relative *Na*/*K* conductivity ratio (0.663) needed for 3 FP’s in the single-*Na*_*v*_-subtype model with Δ*V*_1/2_ = 0 mV. In the voltage range of peak M3 current, the contribution from the *Na*_*v*1.2_ channels is low and does not change much the total current profile. Hence, the upper branch of FP’s is due the contribution of the *Na*_*v*1.6_ channels. **B:** Resting threshold of the mixed models as a function of the *P* parameter. The figure shows *κ*(*P*) = *I*_*THR*_(*P*, Δ*V*_1/2_)/*I*_*THR*_(1,Δ*V*_1/2_) of the mixed model for *T*_*STIM*_ = 100 *μs*. Notice that there is an approximately twofold difference between the thresholds of the M3 and M1 single-*Na*_*v*_-subtype models More than 60% of this difference vanishes for *P* as low as 0.1. The difference becomes minimal for Δ*V*_1/2_ < 4 mV (see also Panel C) or *P* ≥ 0.1. **C:** As the Δ*V*_1/2_ parameter is decreased (from 13 mV down to 0), the M1 contribution becomes larger, thereby reducing the proportion of M3 current needed for 3FP-dynamics. Notice that the proportion of *Na*_*v*1.6_ channels may decrease significantly when Δ*V*_1/2_ < 4 mV. Importantly, this is also about where the transition from M1 to M3 occurs (Δ*V*_1/2_ = *LP*_1_ ≈ -2.94 mV.

The B.D. on Fig 10A shows that, for Δ*V*_1/2_ = 13 mV, the 2 upper FP branches appear at saddle-node bifurcation (see Table 5) for *P* ≈ 0.648, which is close to the minimum relative conductivity (*g*_*Na*_/*g*_*K*_ = 0.663) needed for the single-channel model with Δ*V*_1/2_ = 0 to enter the *M*3 class. In the voltage range of peak *I*_*Na*,∞_[Δ*V*_1/2_ = 0](*V*), the contribution of *I*_*Na*,∞_[Δ*V*_1/2_](*V*) is so low that it does not change much the total *I*_*Na*,∞_ profile. Hence, the upper FP’s rely almost exclusively on the of the *M*3 current contribution. In codimension-2 (see Fig 10C), as Δ*V*_1/2_ decreases, the contribution of *I*_*Na*,∞_[Δ*V*_1/2_](*V*) becomes larger, thereby reducing the proportion of *M*3 current needed to preserve the 3 FP’s. In Fig 10C, the required proportion *P* of *Na*_*v*1.6_ channels (recall the Methods section) decreases abruptly as Δ*V*_1/2_ < 5 mV. Clearly, the latter is due to the intuitive fact that such Δ*V*_1/2_ values mean that the second channel type is itself *tending* to the *M*3 class—a tendency which is completely realized with Δ*V*_1/2_ < **LP1**.

The *relative* resting threshold *κ*(*P*) = *I*_*THR*_(*P*, Δ*V*_1/2_)/*I*_*THR*_(1,Δ*V*_1/2_) of the mixture model is a function of the *P* parameter (*T*_*STIM*_ = 0.1 ms). There is an approximately twofold difference between the thresholds of the *M*3 and *M*1 single-channel models (Fig 10B, see also Fig 2), but more than 70% of this difference vanishes for *P* as low as 0.2. This could be understood by considering the 1D resting version of the mixed model—see Eq (3) with the *m* gates of the two channel subtypes at their saturation values, and all other gates at their resting values. As in section on neuronal excitability, both *I*_*thr*_ and *V*_*thr*_ − *V*_*rest*_ provide measure of excitability. Both *I*_*thr*_ (Fig 10B) and *V*_*thr*_ − *V*_*rest*_ (data not shown) have decreased by more that 70% at *P* = 0.2.

## Discussion

### A generalized model

The model family analyzed in this work stems from the classical Hodgkin-Huxley model (HHM). The latter has been criticized and its very applicability to mammalian neurons has been questioned. Various related HHM optimization directions have been suggested.

Here, we argue that:

- The model family analyzed in this work contains the classical HHM as a special case
- That the validity and applicability of the HHM to mammalian neurons can be achieved simply by picking the Δ*V*_1/2_ parameter value in an appropriate range.

With respect to the first claim above, from Fig 1 the knowledgeable reader can rapidly see that the *quantitative* (and hence the *qualitative*) picture may also be very unlike that of the classical HHM. Namely the interplay between the asymptotic states *y*_∞_(*V*) for the sodium and potassium channels is far more involved: In contrast to the classical HHM, the *h* and *n* gates’ dynamics are *uncoupled*. I.e. the *n* gates can no longer be considered in mere linear correspondence to the *h* gates as is often assumed when working with the HHM and its simplifications such as the Fitzhugh-Nagumo model [45–47].

In the latter *n* = ≈ 1 − *h* lead to a model of reduced dimensionality. Such linear correspondence would however be inadequate over the whole range of Δ*V*_1/2_ parameter variations. For the model considered here, regressing the *n*(*t*) variation during an AP (data not shown) by a polynomial in 1 − *h* requires at least order 2. I.e. there is nonlinear (parabolic) relationship between the *n* and *h* dynamic variables during an AP. Moreover, around the resting state, the *K* ^+^ channels are fully closed (*n*_*r*_ *est* = 0), which is quite unlike the *classical* HHM (where *n*_*r*_ *est* ≈ 0.2). Hence, the resting FP is fully determined by the *leak* parameters (conductance and Nernst potential). This explains the invariance of *V*_*r*_ *est* with Δ*V*_1/2_.

A similar perspective stems also from Fig 4, where the classical HHM corresponds to a narrow range of values for the Δ*V*_1/2_ parameter out of the significantly broader variation range considered by this work.

As to the second claim, in the Results section we dedicated paper space and work effort to demonstrate that indeed essential model family properties, in function of the given Δ*V*_1/2_ parameter value, covary in similar directions as the HHM *optimizations* published in the literature (see also the Summary and Conclusions below).

An general discussion point is also that many of the observed neural dynamics phenomena are nontrivial—i.e. cannot be explained singlehandedly by the model equations. They are the complex outcome from the interplay of the *Na*_*V*_ and *K*_*V*_ channels, their properties and the related multitude of key parameter choices.

### Parametric-model relevance in the light of previous work

How does our parametric analysis of the HH model compare to previous work? Where does it bring new insight and where is it similar? A body of previous work examined bifurcation structure in the Hodgkin-Huxley model. One arguably popular parameter has been *I*_*bias*_. Rinzel and Miller examined the *I*_*bias*_ range for which stable and unstable periodic solutions exist and their findings are qualitatively similar to our own results [48].

The two most relevant other parametric models, which we believe provide parallels and grounds for contrasts are the codimension-2 analysis in the parametric plane *I*_*bias*_ × *E*_*K*_ [49, 50] and the two-dimensional *I*_*Na*,*p*_ + *I*_*K*_ HH-type model [37] with its either high- or low-threshold *I*_*K*_ (i.e. two *E*_*K*_ levels). The latter model is equivalent in many respects to the well-known *I*_*Na*,*p*_ + *I*_*K*_ Morris and Lecar model [51].

The particular interest of these two alternative parameter models is in their choice of “mirror” parameters: The potassium’s Nernst potential *E*_*K*_ is manipulated toward changing the *I*_*K*_ sign-switching point. This may have similar effects to Δ*V*_1/2_ variation, which affects the position of the sodium current window *relative* (closer or farther) to the inversion point of the opposing *K* ^+^ current. However, the two parameters are also *qualitatively* different as the Δ*V*_1/2_ variation affects the onset (and offset) of sodium current through the *dynamics* of *Na* ^+^ channel opening and closing gates.

Both [49, 50] and [37] contain a detailed analysis of the related nonlinear dynamics structure, as well as introduce the “menagerie” of codimension-1 and codimension-2 bifurcation types. This provides an excellent baseline toward interpreting the results of our work. Finally, other previous work (e.g.[52]) examined the variational effects of nominal *g*_*K*_ conductance, relative to nominal *g*_*Na*_. As demonstrated in the Results’ section on mixture models, this may also be qualitatively similar to a Δ*V*_1/2_ variation.

The key difference brought about by Δ*V*_1/2_ variation is that it affects the interplay of sodium and potassium currents not only directly (like compatible *E*_*K*_ or *g*_*K*_ variation) but also *indirectly* through the *V*-dependent temporal dynamics of the HH gates.

## Summary and Conclusions

This work is about bridging theory to practice in an attempt to go all the way from the model mathematics (nonlinear dynamics), through simulations and to the experimental practice. Here we summarize the gist of our key findings.

### Summary of Δ*V*_1/2_-related dynamic properties

In this work we explore the effects of *Na* ^+^ ion-channel properties on neuronal dynamics. Starting from two well-studied channel types ([24, 25]) we observed that a key difference between the *Na*_*v*1.2_ and *Na*_*v*1.6_ types is their half-activation-voltage parameter *V*_1/2_. Hence, we introduced a generalization parameter. This resulted in a parametric family of models exhibiting a continuous variation of sodium current-influx properties.

The introduction of the Δ*V*_1/2_ parameter provides for a comprehensive analysis of neuronal excitability, refractoriness and periodic-stimulation dynamics for a space-clamp model. As seen, this analysis can also be extended further to multiple-compartment models of an entire neuron. In models of higher dimensionality, the spatial distribution of ionic current types and their relative proportions will yield specific spatio-temporal patterns, corresponding to better or worse conditions for AP initiation and propagation. The approach used in this paper can be generalized further to produce these profiles of threshold stimulation-field and stimulation-current values, that lead to successful AP initiation and propagation [36].

The following key effects were determined and explored:

**Continuous** Δ*V*_1/2_ **variation** leads to a bifurcation diagram (Fig 4A) containing a multitude of dynamic regimes. One of the most noteworthy differences is that for Δ*V*_1/2_ ∈ [**LP2**,**LP1**] 3 FP’s coexist. These may also coexist with stable periodic orbits. This means that, depending on the initial conditions, the system may behave as either purely excitable or as an oscillator.
**Absolute vs relative rheobase current:** In the range **LP2** < Δ*V*_1/2_ < **LP1** (the M3 class), the simplified model of Eq (5) can be applied, and −*I*_*ion*,∞_(*V*) has a local minimum −*I*_*rh*_ between *V*_*rest*_ and *V*_*THR*,∞_ (Fig 1C and 1D). Hence an AP can be evoked by applying as little current as the *absolute rheobase* current *I*_*rh*_ for a sufficient amount of time *T*_*STIM*_ (Fig 2).
**In general**, lower Δ*V*_1/2_ values translate into lower threshold current *I*_*THR*_[Δ*V*_1/2_](*T*_*STIM*_) from the resting state (Fig 2).
**The mere FP count** corresponds to nontrivial differences in dynamic organization and its complexity. E.g. supernormality of *I*_*THR*,*S*2_ as a function of *t* − *t*_*up*_, esp. for the lowest Δ*V*_1/2_ (data not shown).
**A complete picture of refractoriness** is given by:
  - the application of the S1-S2 simulation protocol (Fig 7, panel A and Fig 2, panel B). Fig 7 presents the two faces of periodic AP firing in the model. Importantly, and as noted in the Results’ subsection, the *M*1 model sub-class is not only less excitable. Its lowest periodic firing frequency does not get much below 100 *Hz*. Hence, at stimulation durations of about 10 *ms* the models of this sub-class will *already* behave as oscillators. We return to this point in the summary on ‘relative’ refractoriness below.
  - the continuation (Fig A in S1 File) of the bifurcation diagram of Fig 4A, with respect to bias current and periodic regimes (see the Supplementary Results section on the codimension-2 bifurcation structure in the Δ*V*_1/2_ × *I*_*bias*_ parameter plane). Fig A in S1 File demonstrates transitions between pure excitability and oscillation determined by the *I*_*bias*_ and Δ*V*_1/2_ values.
    The automatic-firing region of *I*_*bias*_ variation becomes gradually larger as Δ*V*_1/2_ is increased from 0 to 13 mV (Fig A in S1 File). However the corresponding inter-spike period shows very little variation (see Panel B in Fig 4).
**Absolute** values of *I*_*STIM*_ as a function *t* − *t*_*up*_ and Δ*V*_1/2_ (Fig 7, panel A) imply that “1:1” responses of lower-Δ*V*_1/2_ models can be maintained for either shorter pacing periods or lower stimulation currents.
  However, maintaining ∀Δ*V*_1/2_ a similar proportion of *I*_*STIM*_ relative to the resting threshold *I*_*THR*,0_[Δ*V*_1/2_](*T*_*STIM*_) (see also Fig 2), Fig 7B predicts that the higher-Δ*V*_1/2_ models would be the first to behave as oscillators as *T*_*STIM*_ is increased.
**’Relative’ refractoriness** may be the consequence of more complex dynamic organization. Thus low Δ*V*_1/2_ means higher excitability and the existence of an absolute rheobase (Figs 1C and 2). However, the higher number of FP’s implies more features and—hence, constraints in phase space (Fig 5).
  The the semi-transparent surface in Fig 7A represents the (1D) dependence of resting threshold *I*_*STIM*_ on Δ*V*_1/2_ for *T*_*STIM*_ = 100 *μs*, applying ∀Δ*V*_1/2_ a 150% *safety factor* for robust AP-triggering—i.e.
  $$\begin{array}{c} I_{STIM}=1.5\times I_{THR,0}\left[\Delta V_{1/2}\right]\left(T_{STIM}\right) \end{array}$$
  This is intersects into the main 3D surface, which shows the minimum 1:1 pacing *I*_*STIM*_ currents as a function of Δ*V*_1/2_ and the pacing interval *τ*_11_ (the black-yellow line in Fig 7A).
  For *I*_*STIM*_ close to the *I*_*THR*_ value (for a given model), the *M*1 sub-class is paced at a faster frequency. Such higher ‘relative’ refractoriness is also clearly present in Fig 7B where the longest automatic-firing period is substantially shorter for Δ*V*_1/2_ = 13 mV, than it is for Δ*V*_1/2_ = 0 mV.
  Sometimes this may not be desired (see the Discussion points about a large frequency-encoding range, above).
  However, when *I*_*STIM*_ is much above the *I*_*THR*_ value, the trend can be completely reversed, given that the *M*3 model sub-class is both more excitable and has a very large frequency range (Fig 7B and Fig D.B in S1 File). The higher ‘relative’ refractoriness of the *M*3 sub-class may be in part due to the *h* gates’ dynamics, since the position of the resting FP (*V*_*rest*_) does not vary much with Δ*V*_1/2_. Hence, the *h* gates’ may need a longer-lasting recovery phase of the membrane to regain excitability (Fig 3A and 3B).
**Simplified models** such as the ones relying on the concept of “hidden structure” (a generalization of the PTC concept for non-linear oscillators, data not shown) capture the essential parts of the complex dynamics—such as the invariance of the evoked AP and the latency dependence on *I*_*STIM*_ compared to *I*_*THR*_.

### On the need for a methodology to study and predict neuronal dynamics determined by ion-channel distributions

Nonlinear dynamics mechanisms may explain some experimental observations in a predictive way. A methodology to study the effect of ion-channel distributions on neuronal dynamics, excitability and refractoriness holds promise for experimental neuroscience practice (e.g. [53]).

Hence, such modeling and analysis are worth pursuing. Functional electric stimulation is at the threshold of a new realm of possibilities. The paradigm is rapidly shifting from classical work ([54, 55]), as rapid stimulation (typically through trains of ∼ 300 Hz) to evoke single AP’s in isolated neurons is largely suboptimal.

Recent experimental ICMS work (e.g. [53]) confirmed the modeling predictions (e.g. [22, 25, 26]) that AP’s are evoked almost exclusively from axonal targets, even if this results antidromic AP propagation. Due to refractoriness, an outcome of ‘ad hoc’ high-frequency stimulation trains may be depression instead of activation. A given electrode(s) geometry and relative position with respect to the targeted excitable tissue will imply a given *Na*_*v*_ channel density (as the latter depends on the underlying cell(s) morphology). From our analysis, it may be inferred that particular positions and stimulation protocols may be appropriate for a given desired outcome.

Together with critical aspects such as the long-term compatibility, functional visual prosthetics require effective and reliable (1:1) stimulation of select (precisely targeted) neural sub-populations. Such stimulation not only uses optimally little energy (from an optimal-control point of view) and has minimum undesirable side-effects (e.g. tissue damage or involuntary saccades, [56]). Selective stimulation with lowest possible currents also means higher possibility for conveying information through visual percepts and specific visual features: e.g. oriented lines conveying shape or colored stimuli versus bright but color-less and feature-less phosphenes ([57]). Finally, a very large impact on the type of possibilities is determined by the size, type and number of the stimulation electrodes that are used, as well as by the precision of their guidance to their neuronal targets. We demonstrated here that with reasonably high *I*_*STIM*_ values the limit periods of 1:1 pacing may be as short as ∼ 5 ms. Hence stimulation train frequency can be as high as 200 Hz. The latter result is consistent with the experimentally observed fact that past such frequencies the subjective visual percept reaches a plateau, beyond which it starts getting weaker. This is suggestive that the neuronal targets of stimulation will respond with activation ratios lower than unity to such ‘overdrive’.

Theory needs to build bridges to practice—from modeling (mathematics, nonlinear dynamics, simulations) to the applications such as functional electric stimulation.

## Appendix A: Supplementary Results

### The effect of Δ*V*_1/2_ on automaticity

Applying the *S*1/*S*2 protocol to study refractoriness implicitly assumes that the system does not reach an automatic regime—i.e. produce multiple AP’s during either of the *S*1 or *S*2 stimuli. For brief stimulation, the system reverts to the non-stimulated dynamics during re-polarization. However, as *T*_*STIM*_ increases, new attractors associated to long stimulation cannot be ignored—the system may now approach them. Hence, the outcome depends on the duration of the transient trajectories leading to these attractors. The latter are revealed by the bifurcation structure as a function of a constant bias current parameter (*I*_*bias*_) Such current is known to change the attractors of the system (e.g. [37], Chapter 4).

Figs B and C in S1 File present examples of the rich bifurcation structure created by a constant stimulation current *I*_*bias*_ for different values of Δ*V*_1/2_. As Δ*V*_1/2_ decreases, the FP curve changes from a monotonous increasing single-branch (Fig C in S1 File, top panel: Δ*V*_1/2_ = 13 mV) to a three-branch organization (e.g Δ*V*_1/2_ = 3 and -8 mV). The stability of the hyperpolarized FP is lost through a *subcritical Hopf* bifurcation (see Table 5). The most depolarized FP also becomes stable by a supercritical Hopf (HB) at a very high value of bias current. High amplitude stable PO’s also exist in an *I*_*bias*_ range specific to each Δ*V*_1/2_. These stable cycles are created by a saddle-node of cycles bifurcation (see Table 5), which produces also unstable cycles. The unstable PO’s may connect with the subcritical HB (of the lower branch), as in the case of Δ*V*_1/2_ = 13 mV. The interaction of the unstable PO’s with the low-Δ*V*_1/2_ (yielding a three-branch FP structure) is interesting. They may end through a *homoclinic* bifurcation (see Table 5), when hitting the middle FP branch, as in the case of Δ*V*_1/2_ = 3 mV. In the latter case, the unstable cycles created at the **HB1** also disappear through a similar homoclinic bifurcation, but at a different (higher) bias current. Hence, different situations are possible depending on the values of Δ*V*_1/2_ and *I*_*bias*_: a unique stable FP; bi-stability between a stable FP and a stable PO, which in turn are separated by an unstable cycle and/or two unstable fixed points; a unique stable cycle with one or three unstable fixed points (see the zoomed-inset in Fig C in S1 File, the respective phase-plane trajectories, corresponding to the “menagerie” of possibilities, are shown in Figs H,I and J in S1 File).

How long does the transient take to reach a stable cycle from the resting state? Some clue is provided by the cycle period. As seen in Fig D.B in S1 File, the period is a monotonously decreasing function of *I*_*bias*_ with a minimum well below 5 ms.

### The codimension-2 bifurcation structure in the Δ*V*_1/2_ × *I*_*bias*_ parameter space

*I*_*bias*_ stands for the magnitude a constant current injected into the modeled compartment and is known as *bias* current.

Already from the phase portrait of typical sub- and supra-threshold trajectories (Fig 5) one gets a pretty colorful idea about the dynamic-structure richness of the parametric HH-type model. Consistently to [49, 50], in this analysis we found a very rich a codimension-2 bifurcation structure.

Fig A in S1 File presents the full picture of the rather complex codimension-2 bifurcation structure in the (Δ*V*_1/2_, *I*_*bias*_) parameter-plane (see also Fig B in S1 File). The main features are:

**Cyan trace** shows the codimension-2 positions of the two Hopf bifurcations **HB1** and **HB2** as a function of the model parameters. Note that for the low Δ*V*_1/2_ there is an only-apparent “collision” of the two (but see also Fig D.A in S1 File). While for very high Δ*V*_1/2_ the two HB’s indeed coalesce, which in codimension-2 is known as *Generalized Hopf* (see Table 5). This case is further illustrated by Fig F in S1 File.
  An important feature here is that, slightly before the two HB’s indeed coalesce, their type changes from *subcritical* to *supercritical*, which is accompanied by the disappearance of two extra branches of stable/unstable PO’s (see Fig F in S1 File and **SNC5** in Fig B.A in S1 File).
**Dashed-black lines** These illustrate the parameter-range for the saddle-nodes **LP1** and **LP2** through which are created the 2 additional FP branches (saddle in the middle branch and unstable center/focus in the top branch of the B.D.’s, see Fig 4A and Fig C in S1 File). Here, there are also 2 related codimension-2 bifurcation phenomena. First, the two LP’s coalesce in what is known as *Cusp* (see Table 5). Second, for low-enough Δ*V*_1/2_ **LP1** coalesces with **HB1** through a codimension-2 bifurcation known as *Takens-Bogdanov* (see Table 5).
**Red and blue lines** the Δ*V*_1/2_ × *I*_*bias*_ range of stable/unstable PO’s determined by the saddle-nodes for cycles **SNC1** and **SNC2** (the red locus) and **SNC3** and **SNC4** (the blue locus, see also Fig B in S1 File)
  Notice that outside (below and over) the HB area, there is at least *bi-stability* of a stable fixed point with stable PO (or *multi-stability* between the stable FP and two stable PO’s, see also Fig B.A in S1 File and the case of Δ*V*_1/2_ = 3 *mV* in Fig C in S1 File). For very large *I*_*bias*_ currents (beyond the blue trace) only the stable depolarized fixed point remains (apart when the island described next exists).
  For very large Δ*V*_1/2_ the stable/unstable PO’s between **SNC1** and **SNC2** detach from the FP’s locus to form an *island* (see Fig G in S1 File).

## Supporting Information

S1 File Supplementary Figures. **Fig A The Δ*V*_1/2_ × *I*_*bias*_ parameter plane, bistability and automatic regimes** Bifurcation structure in the Δ*V*_1/2_ × *I*_*bias*_ parameter plane Cyan trace: the Hopf bifurcations (HB) as a function of the *I*_*bias*_ and Δ*V*_1/2_ model parameters (see also Fig D.A). Dashed-black lines: Existence/creation parameter-range of the 2 additional fixed points (saddle-node in the middle branch, and unstable center/focus in the top branch of the B.D.’s, see Fig 4A and 4C) Red and blue lines: the Δ*V*_1/2_ × *I*_*bias*_ range of stable/unstable PO’s (see also Fig G) Notice that outside (below and over) the HB area, there is bistability with a stable fixed point, while for very large *I*_*bias*_ currents (beyond the blue trace) only the stable depolarized fixed point remains (see also Fig C). For very large Δ*V*_1/2_ the stable/unstable PO’s between **SNC1** and **SNC2** detach from the FP’s locus to form an *island* (see also Fig F and G)
**Fig B The Bifurcation Glossary (Codimension 1 and possibly 2) terms illustrated for two Δ*V*_1/2_ cases** **black lines:** stable FP’s. They lose (or recover) stability via Hopf Bifurcations **red lines:** unstable FP’s. In A, unstable FP’s appear (or disappear) via Saddle node (SN) bifurcations at **LP1** and **LP2**. **blue lines:** Minimum and Maximum voltage of stable periodic orbits. These can appear (or disappear) through SNC bifurcations or supercritical HB’s (e.g. the low amplitude stable cycles in B). **cyan lines:** Minimum and Maximum voltage of unstable periodic orbits. These can appear (or disappear) through SNC bifurcations or subcritical HB (e.g. **HB2** in A), or homoclinic bifurcation (e.g. the lower open ends in A).
**Fig C BD’s for a set of Δ*V*_1/2_ values—see legend, by stable PO (SPO) color** **Inset:** Zoom in of the Δ*V*_1/2_ = 3 *mV* case for *I*_*bias*_ ∈ [7, 14]*μA*/*cm* ^2^
**Fig D Combined BD’s and periods of the high-amplitude cycle—in the Δ*V*_1/2_ × *I*_*bias*_ parameter space** **A:** Combined BD’s **B:** max. cycle-periods as a function of Δ*V*_1/2_ × *I*_*bias*_ parameters (B) Note that the periods of the low-amplitude cycles (around *SNC*3 and *SNC*4) have not been continued.
**Fig E BD’s for a set more of Δ*V*_1/2_ values (see the panel legends, by SPO color)** (see also Fig C)
**Fig F Generalized Hopf in codimension-2: Collision of two Hopf Bifurcations (HB)** As the two Hopf’s become closer in parameter space, one unstable PO (UPO) is lost. Moreover, the HB type changes from *sub*-critical to *super*-critical (see also Fig G)
**Fig G The creation of an *island* in codimension-2** A little before the 2 HB’s collide, the small-amplitude SPO detaches from the UPO. The latter forms an island together with the large-amplitude SPO. (see also Fig F)
**Fig H Very complex dynamic organization for** Δ*V*_1/2_ = 3 *mV* **and** *I*_*bias*_ = 9.5 *μA*/*cm* ^2^ **A:** Temporal evolution of membrane voltage *V* for the SPO (magenta, green trace on Panel C) and the UPO (black, thick cyan trace on Panel C). **B:** Gate states dynamics for the SPO (green trace on Panel C). Notice the very limited range of the *n* gate’s variation. **C:** The unstable FP invariant directions yield transient trajectories which converge mostly to the SPO. This is due to the UPO (thick cyan trace). The transient trajectories starting at the UPO’s single unstable invariant direction converge respectively to the resting FP and to the SPO (thinner dashed cyan traces). See also Fig H.A
**Fig I Examples of dynamic objects (phase-space trajectories) corresponding to bifurcations in codimension-2.** *I*_*bias*_ variation for Δ*V*_1/2_ = 3 *mV* results in a complex organization (see the B.D. in Fig H—middle row and Inset). The figure presents the rich diversity of dynamic objects (e.g. heteroclinic, homoclinic, stable and unstable periodic orbits) by simulating them for 3 values of the *I*_*bias*_ parameter: 7.44, 7.49 and 9.068 *μA*/*cm* ^2^. Heteroclinic (dashed red), homoclinic (blue), stable (green) and unstable (cyan and black, Panel A only) periodic orbits
**Fig J An unstable limit cycle as a phase-space separatrix** **A:** Due to the existence of an UPO (cyan), all trajectories (black and red) initiated on the unstable FP’s (red triangle) invariant directions deviate toward the resting FP (blue circle) and not to the SPO (green). Incidently (and like in the B.D.’s on Fig F.C), the UPO’s own invariant direction yields 2 transient trajectories (data not shown) which converge respectively to the resting FP *and* to the SPO. **B:** An UPO is absent here unlike Panel A. Hence, the same initial conditions as in Panel A yield transient trajectories half of which (2 out of 4) converge to the SPO.
(PDF)
