# Supplementary material for: From Squid to Mammals with the HH Model through the Nav Channels’ Half-Activation-Voltage Parameter
Source: PLoS One. 2015 Dec 2;10(12):e0143570. doi: 10.1371/journal.pone.0143570 (PMC4667926; doi:10.1371/journal.pone.0143570)
Supplement: S1 File — Fig A The ΔV1/2 × Ibias parameter plane, bistability and automatic regimes Bifurcation structure in the ΔV 1/2 × I bias parameter plane Cyan trace: the Hopf bifurcations (HB) as a function of the I bias and ΔV 1/2 model parameters (see also Fig D.A). Dashed-black lines: Existence/creation parameter-range of the 2 additional fixed points (saddle-node in the middle branch, and unstable center/focus in the top branch of the B.D.’s, see Fig 4A and 4C) Red and blue lines: the ΔV 1/2 × I bias range of stable/unstable PO’s (see also Fig G) Notice that outside (below and over) the HB area, there is bistability with a stable fixed point, while for very large I bias currents (beyond the blue trace) only the stable depolarized fixed point remains (see also Fig C). For very large ΔV 1/2 the stable/unstable PO’s between SNC1 and SNC2 detach from the FP’s locus to form an island (see also Fig F and G) Fig B The Bifurcation Glossary (Codimension 1 and possibly 2) terms illustrated for two ΔV1/2 cases black lines: stable FP’s. They lose (or recover) stability via Hopf Bifurcations red lines: unstable FP’s. In A, unstable FP’s appear (or disappear) via Saddle node (SN) bifurcations at LP1 and LP2. blue lines: Minimum and Maximum voltage of stable periodic orbits. These can appear (or disappear) through SNC bifurcations or supercritical HB’s (e.g. the low amplitude stable cycles in B). cyan lines: Minimum and Maximum voltage of unstable periodic orbits. These can appear (or disappear) through SNC bifurcations or subcritical HB (e.g. HB2 in A), or homoclinic bifurcation (e.g. the lower open ends in A). Fig C BD’s for a set of ΔV1/2 values—see legend, by stable PO (SPO) color Inset: Zoom in of the ΔV 1/2 = 3 mV case for I bias ∈ [7, 14]μA/cm 2 Fig D Combined BD’s and periods of the high-amplitude cycle—in the ΔV1/2 × Ibias parameter space A: Combined BD’s B: max. cycle-periods as a function of ΔV 1/2 × I bias parameters (B) Note that the periods of the low-amplitude cycles (around SNC3 [file pone.0143570.s001.pdf]

Supporting Information Figures

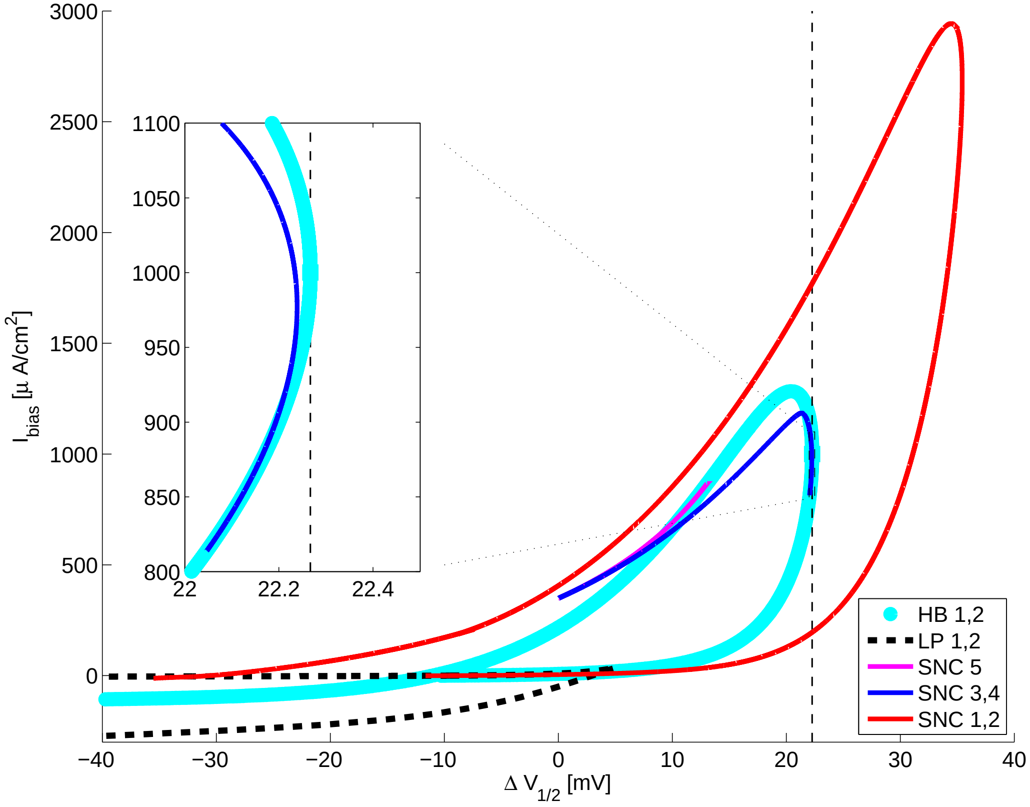

Fig.A The $\Delta V_{1/2} \times I_{bias}$ parameter plane, bistability and automatic regimes

Bifurcation structure in the $\Delta V_{1/2} \times I_{bias}$ parameter plane Cyan trace: the Hopf bifurcations (HB) as a function of the I_{bias} and $\Delta V_{1/2}$ model meta-parameters (see also Fig.S4A). Dashed-black lines: Existence/creation parameter-range of the 2 additional fixed points (saddle-node in the middle branch, and unstable center/focus in the top branch of the B.D.'s, see Fig.**??**B and Fig.3) Red and blue lines: the $\Delta V_{1/2} \times I_{bias}$ range of stable/unstable PO's (see also Fig.S7) Notice that outside (below and over) the HB area, there is bistability with a stable fixed point, while for very large I_{bias} currents (beyond the blue trace) only the stable depolarized fixed point remains (see also Fig.3). For very large $\Delta V_{1/2}$ the stable/unstable PO's between **SNC1** and **SNC2** detach from the FP's locus to form an *island* (see also Fig.S6 and S7)

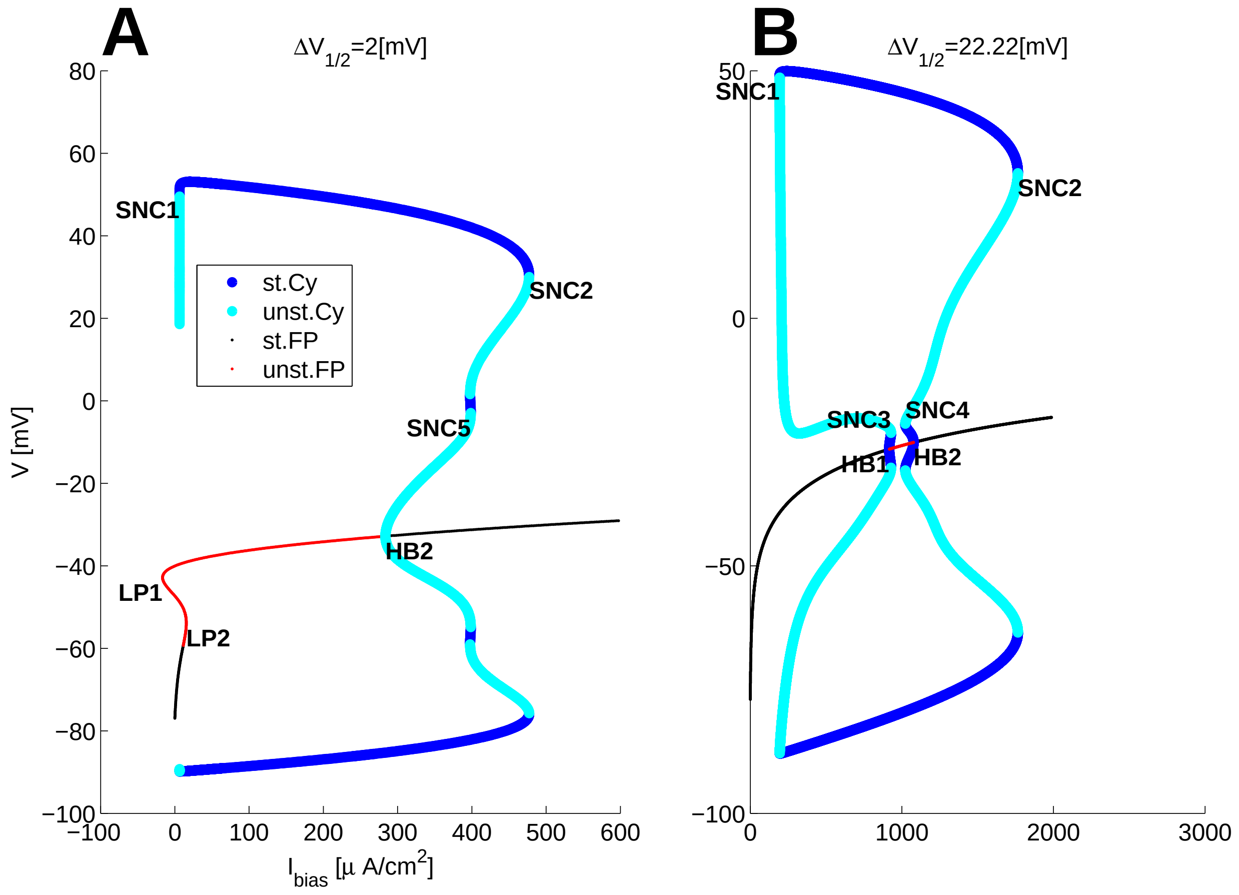

Fig.B The Bifurcation Glossary (Codimension 1 and possibly 2) terms illustrated for two $\Delta V_{1/2}$ cases

black lines: stable FP's. They lose (or recover) stability via Hopf Bifurcations **red lines:** unstable FP's. In A, unstable FP's appear (or disappear) via Saddle node (SN) bifurcations at **LP1** and **LP2**. **blue lines:** Minimum and Maximum voltage of stable periodic orbits. These can appear (or disappear) through SNC bifurcations or supercritical HB's (e.g. the low amplitude stable cycles in B). **cyan lines:** Minimum and Maximum voltage of unstable periodic orbits. These can appear (or disappear) through SNC bifurcations or subcritical HB (e.g. **HB2** in A), or homoclinic bifurcation (e.g. the lower open ends in A).

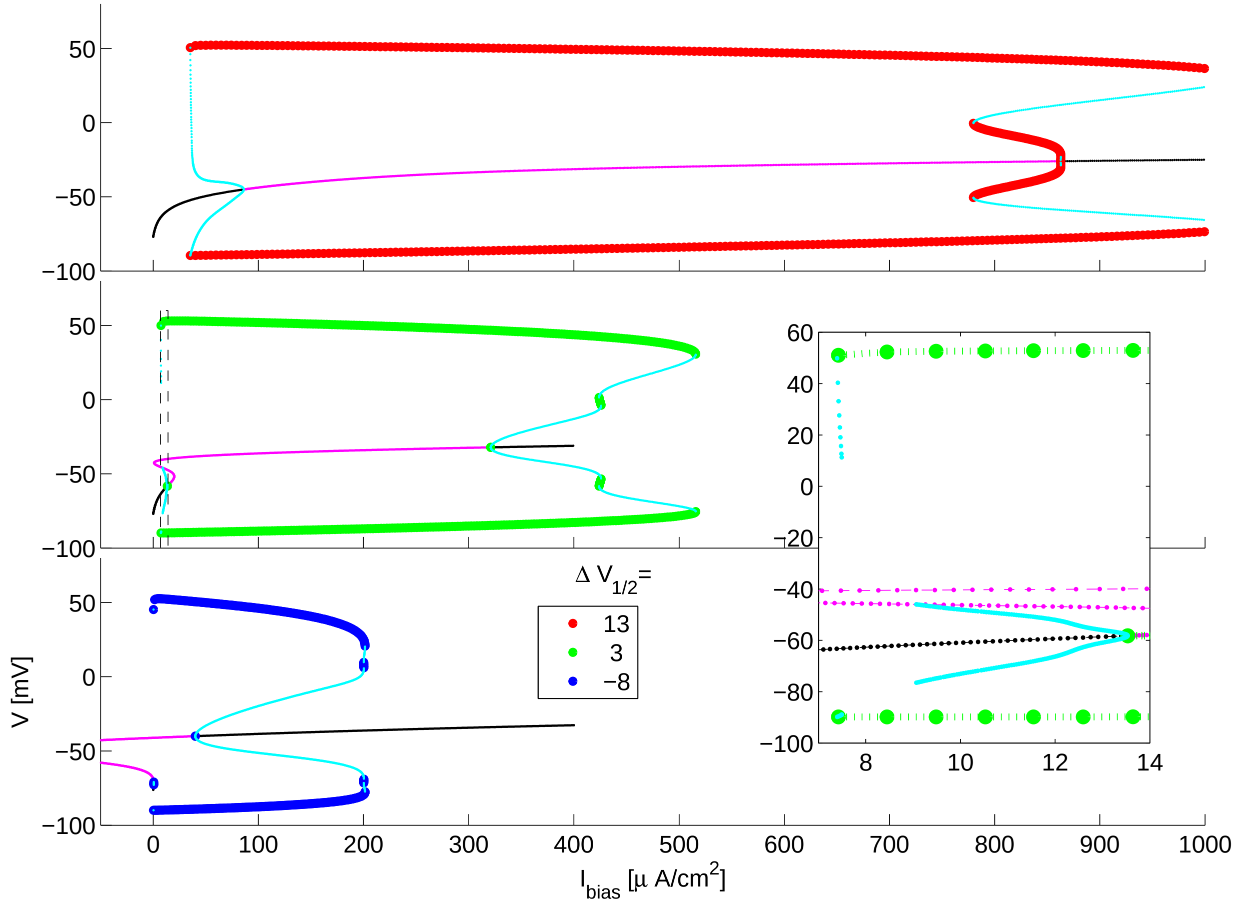

Fig.C BD's for a set of $\Delta V_{1/2}$ values (see legend, by SPO color)

Inset: Zoom in of the $\Delta V_{1/2} = 3\ mV$ case for $I_{bias} \in [7, 14] \mu A/cm^2$

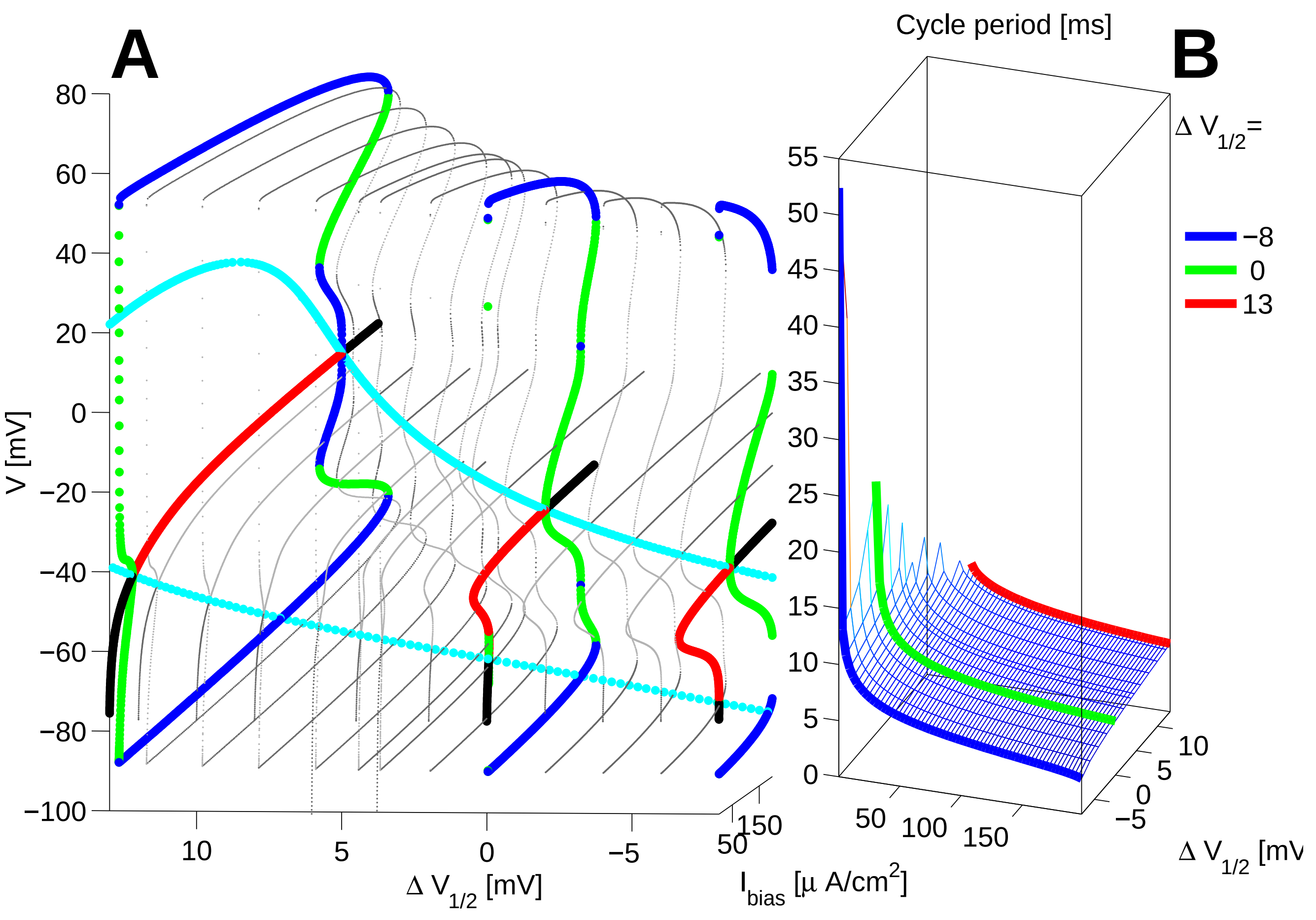

Fig.D Combined BD's and cycle-periods in the $\Delta V_{1/2} \times I_{bias}$ parameter space

A: Combined BD's **B:** max. cycle-periods as a function of $\Delta V_{1/2} \times I_{bias}$ parameters (B)

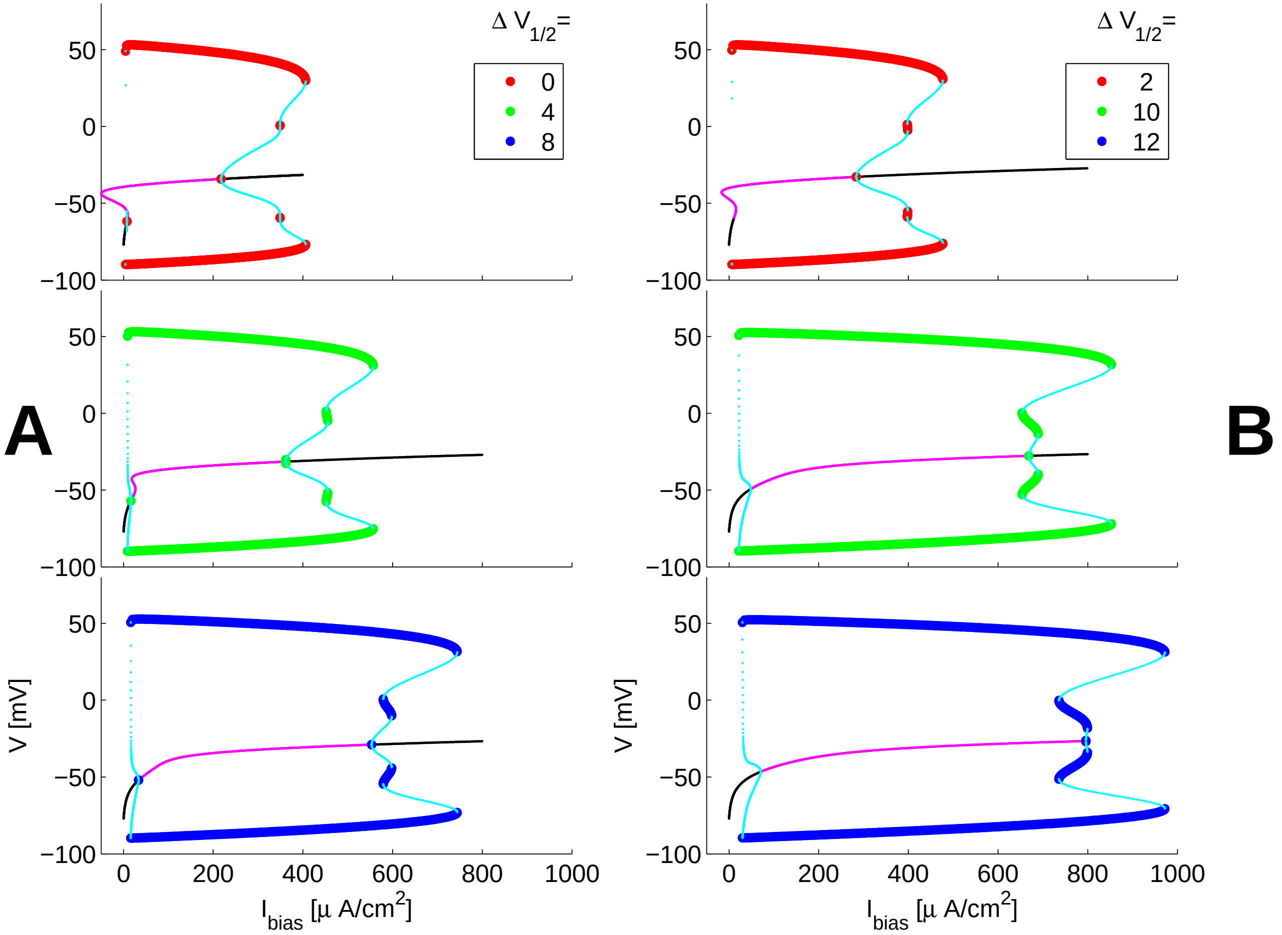

Fig.E BD's for a set more of $\Delta V_{1/2}$ values (see the panel legends , by SPO color)

(see also Fig.3)

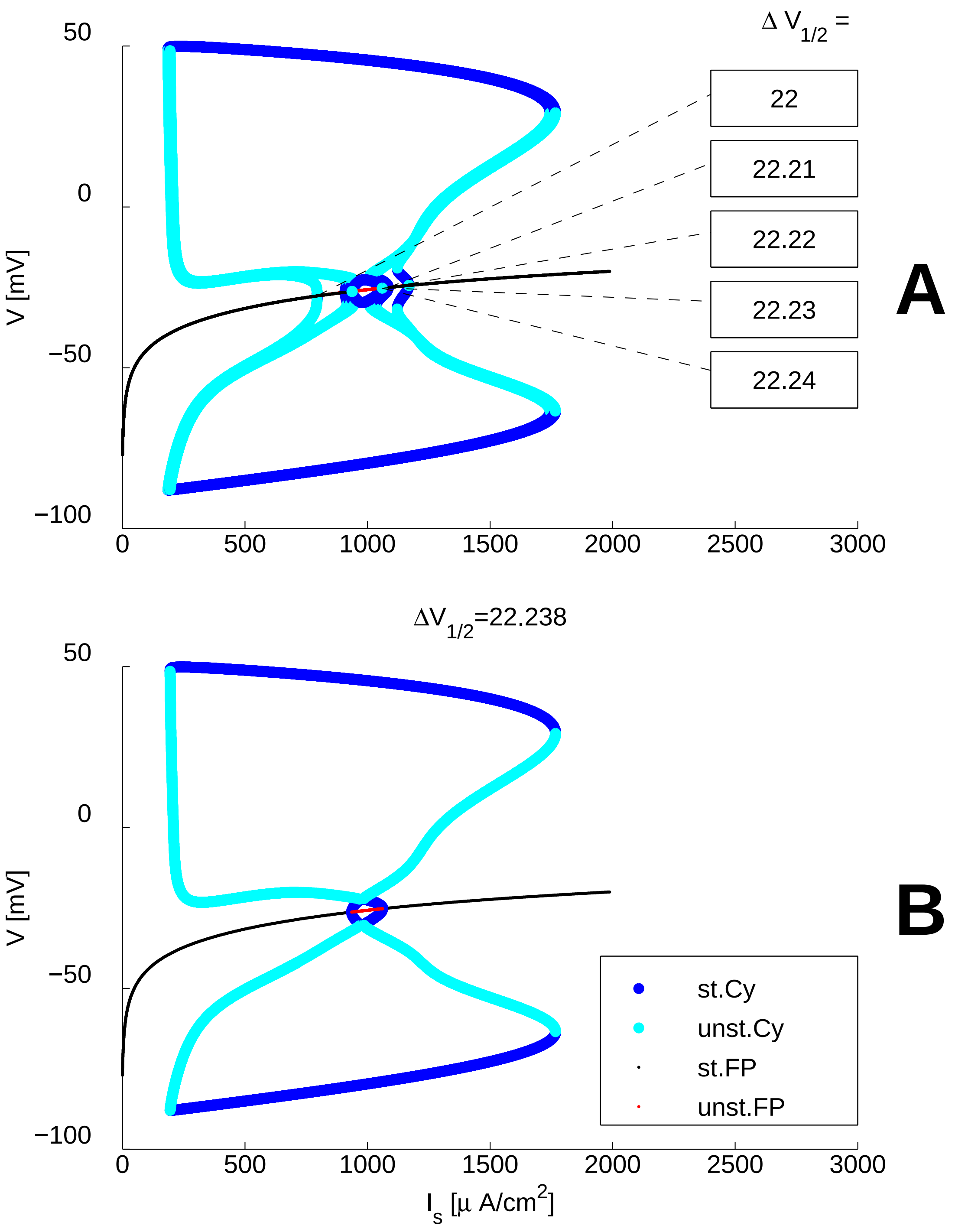

Fig.F Generalized Hopf in codimension-2: Collision of two Hopf Bifurcations (HB)

As the two Hopf's become closer in parameter space, one UPO is lost. Moreover, the HB type changes from *sub*-critical to *super*-critical (see also Fig.S7)

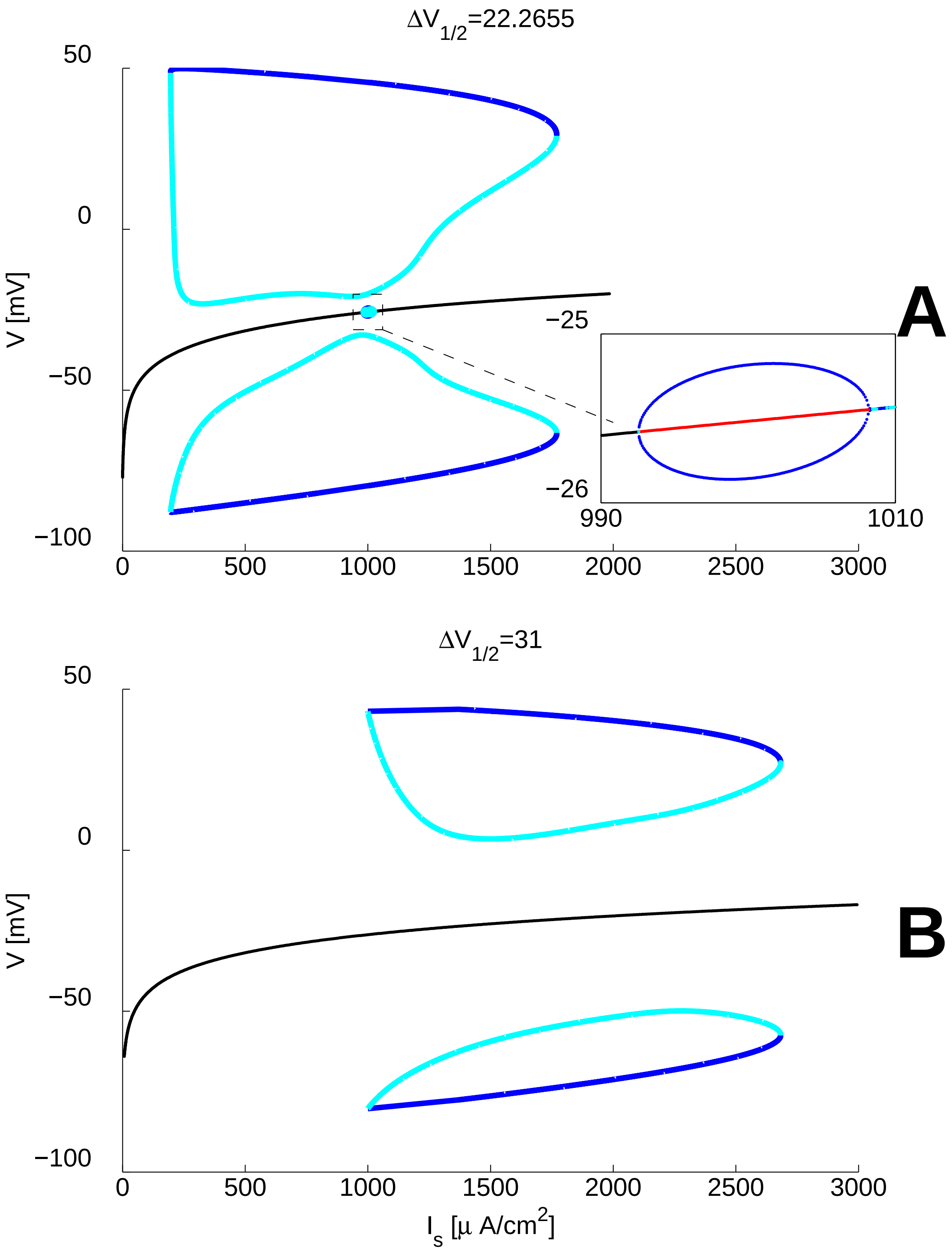

Fig.G The creation of an *island* in codimension-2 A little before the 2 HB's collide, the small-amplitude SPO detaches from the UPO. The latter forms an island together with the large-amplitude SPO. (see also Fig.S6)

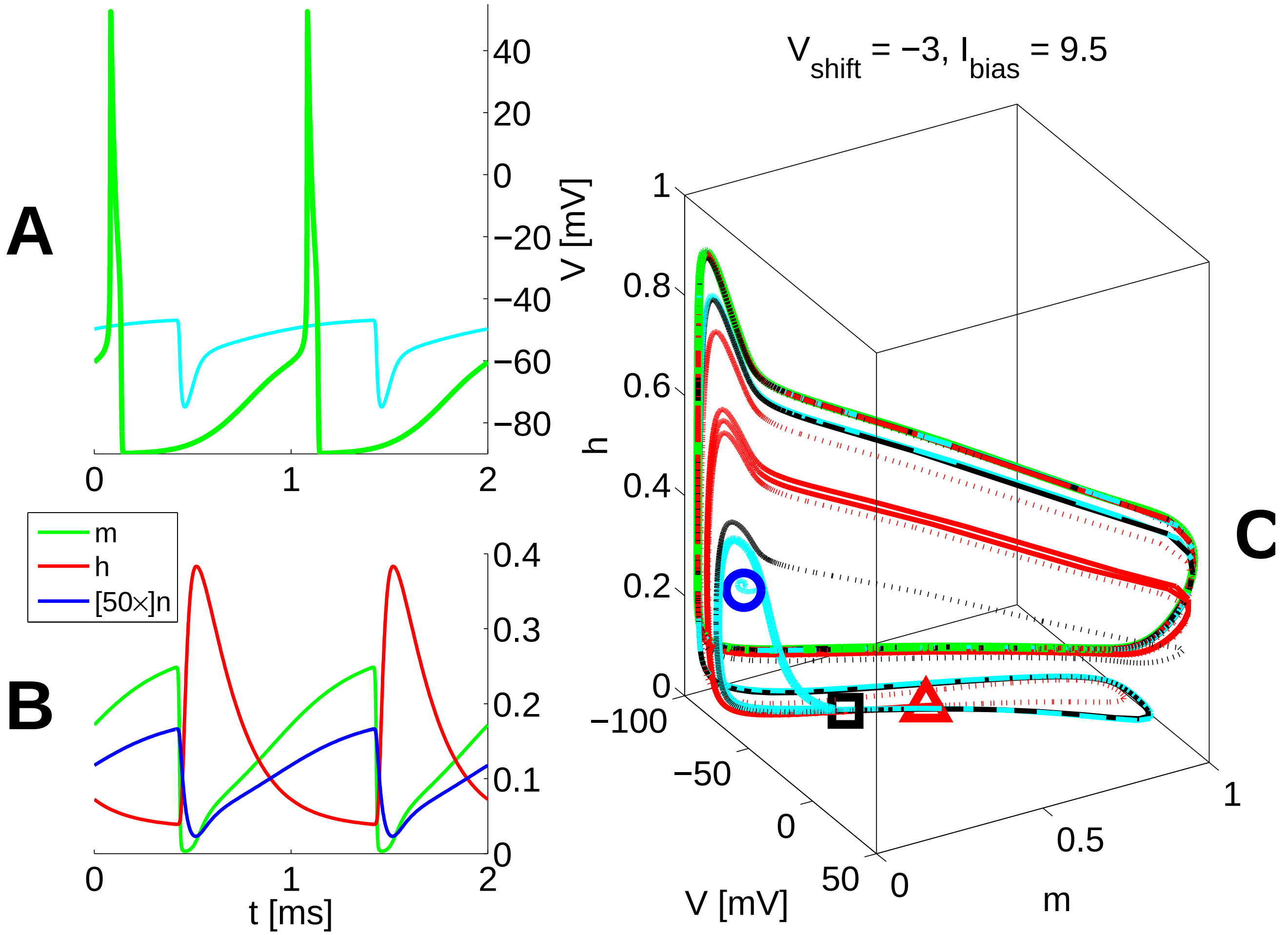

Fig.H Very complex dynamic organization for $\Delta V_{1/2} = 3\ mV$ and $I_{bias} = 9.5\ \mu A/cm^2$

A: Temporal evolution of membrane voltage V for the SPO (magenta, green trace on Panel C) and the UPO (black, thick cyan trace on Panel C). **B:** Gate states dynamics for the SPO (green trace on Panel C). Notice the very limited range of the n gate's variation. **C:** The unstable FP invariant directions yield transient trajectories which converge mostly to the SPO. This is due to the UPO (thick cyan trace). The transient trajectories starting at the UPO's single unstable invariant direction converge respectively to the resting FP and to the SPO (thinner dashed cyan traces). See also Figs.S8A

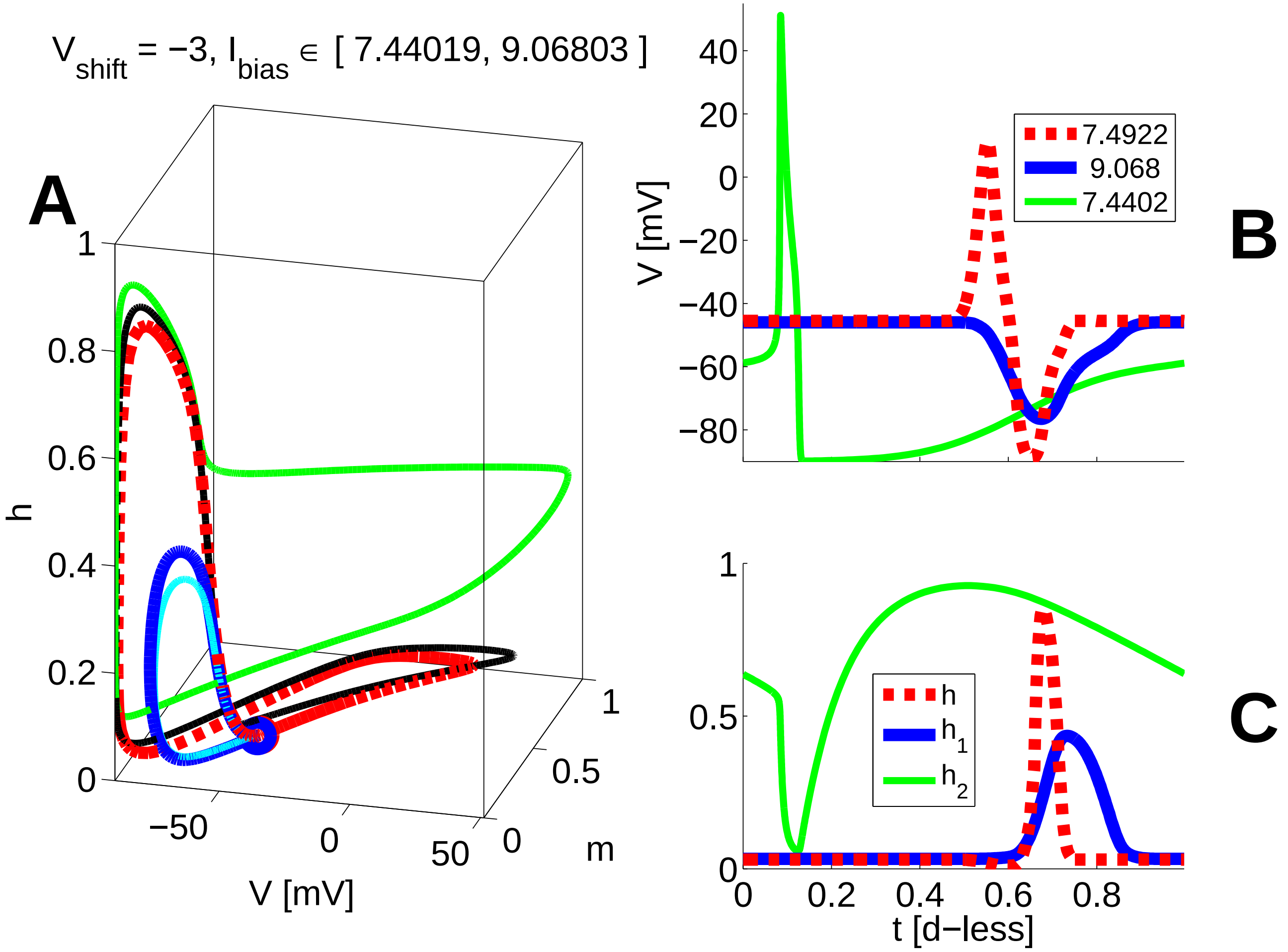

Fig.I I_{bias} **for the B.D.'s limit cases**

Heteroclinic (dashed red), homoclinic (blue), stable (green) and unstable (cyan and black, Panel A only) periodic orbits

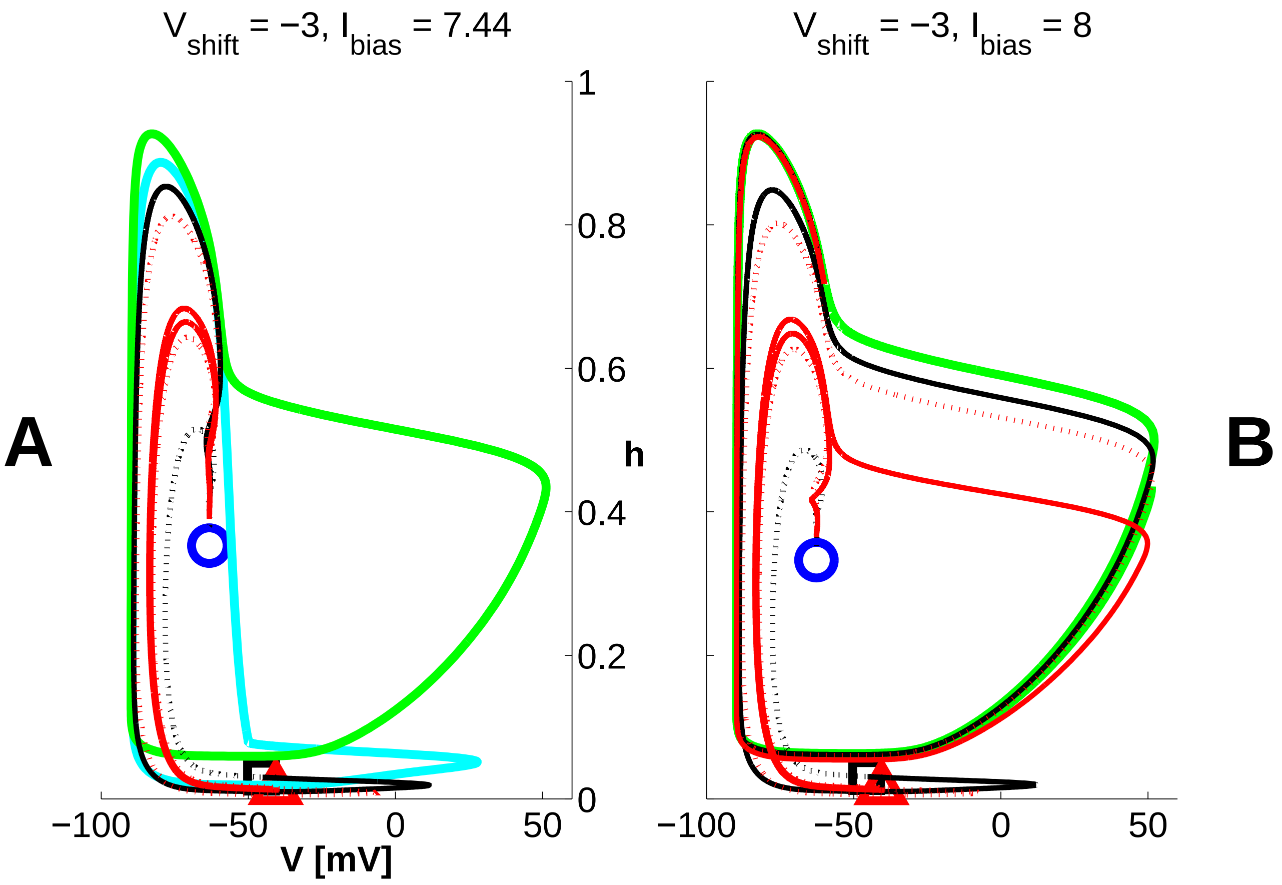

Fig.J An unstable limit cycle as a phase-space separatrix

A and B: Notice how the UPO in the Panel-A case makes all trajectories initiated at unstable FP invariant directions converge onto the resting FP and not to the SPO. Incidently and like in Figs.S6C, the UPO's own invariant direction yields 2 transient trajectories which converge respectively to the resting FP *and* to the SPO.
